# Life histories of Antarctic incirrate octopods (Cephalopoda: Octopoda)

**DOI:** 10.1371/journal.pone.0219694

**Published:** 2019-07-11

**Authors:** Richard Schwarz, Henk-Jan Hoving, Christoph Noever, Uwe Piatkowski

**Affiliations:** 1 GEOMAR Helmholtz Centre for Ocean Research Kiel, Evolutionary Ecology of Marine Fishes, Kiel, Germany; 2 Department of Biological Sciences, University of Bergen, Bergen, Norway; The Chinese University of Hong Kong, CHINA

## Abstract

As a general trend in the life history of marine organisms, species inhabiting cold waters have reduced growth rates and increased lifespans. Studies based on egg sizes and brooding times of deep-sea and polar octopods support this hypothesis, but empirical data on growth are still scarce. To test the hypothesis that octopods inhabiting cold waters (< 3°C) live longer than temperate and warm water species, this study investigated size-at-age, maturation and growth rates in incirrate Antarctic octopods. Octopod age was estimated via the interpretation and quantification of beak growth increments, which in shallow water octopods have been validated to be formed on a daily basis. Specimens from the families Megaleledonidae (*Adelieledone* spp., *Pareledone* spp. and *Megaleledone setebos*) and Enteroctopodidae (*Muusoctopus rigbyae*) were collected on the shelf and slope regions off the Antarctic Peninsula during a cruise in 2012. Examined specimens included early juveniles to animals in advanced maturity. The total number of growth increments ranged from 192–599 in *Pareledone aequipapillae* (body mass [BM] 2–109 g), 182–431 in *Pareledone charcoti* (BM 5–124 g), 98–906 in *M*. *setebos* (BM 10–6000 g) and 207–425 in *M*. *rigbyae* (BM 24–256 g). After the cruise, eleven specimens of *P*. *charcoti* were kept alive in captivity for more than 12 months and these animals had 219–364 growth increments, suggesting that increment formation in this species takes longer than one day. The complex population structure (size, age and maturity range) of the specimens that were captured during a relatively short time, the number of beak increments quantified, and the preliminary validation observations indicate that Antarctic octopods do not deposit increments daily, and may have lifespans exceeding 3 years. These findings corroborate the general trend that cold water molluscs have a longer lifespan than their warm water relatives.

## Introduction

The marine benthic invertebrate fauna of the Southern Ocean is highly diverse and adapted to the harsh environmental conditions of high latitudes [1]. Antarctic invertebrates evolved in such a way that these organisms are able to cope with extremely low temperatures and highly seasonal productivity [2,3]. The permanently cold water (-2 to 3°C) slows down physiological processes in Southern Ocean marine ectotherm invertebrates, resulting in increased lifespans in echinoderms, molluscs and crustaceans compared to their temperate relatives [4–6]. As an example, the crangonid shrimp *Notocrangon antarcticus* (Pfeffer, 1887) from the Weddell Sea lives in a temperature range of -2 to 0.4°C with a lifespan of 8 to 10 years, while *Crangon crangon* (Linnaeus, 1758) from the North Sea lives for approximately three years at temperatures of 7 to 16°C [5]. Life history theory predicts that animals with increased lifespans mature relatively late in life and invest more energy in fewer but larger offspring. These traits increase survival in the hatchlings, and compensate for the mortality risks of a longer embryonic and juvenile period [7].

Many benthic molluscs from the Southern Ocean follow the trend described above and typically invest in the production of well-developed benthic juveniles, avoiding the high mortality rates experienced by planktotrophic larvae which are common in temperate species [4,8]. Gastropod species from the Weddell Sea can produce eggs that are four times larger than those of their temperate relatives, whilst embryogenesis can be 30 times as long, with some species taking as much as 25 months to hatch [9]. The large egg cases (52 mm) produced by the Antarctic nudibranch *Bathydoris hodgsoni* (Eliot, 1907) are estimated to require up to 10 years for their complete embryonic development. The hatchlings of this species are 29 mm and among the largest hatchlings of molluscs [10].

Southern Ocean cephalopods are also known to produce fewer and larger offspring than their tropical and temperate counterparts [11]. Benthic incirrate octopods are the most diverse cephalopod group in the region, comprising 27 of the 54 cephalopod species identified to date [12]. The majority of octopod species endemic to the Southern Ocean belong to the family Megaleledonidae, which also contains deep-sea taxa that occur in other ocean regions [13]. On the shelf regions of the Antarctic Peninsula and South Shetland Islands, the most common octopod species are from the genus *Pareledone* (10 species), the genus *Adelieledone* (three species) and the giant Antarctic octopus *Megaleledone setebos* (Robson, 1932) [14–19].

Antarctic octopods are important components in the diets of demersal fishes, southern elephant seals and Weddell seals [20–22]. However, knowledge about their feeding ecology and trophic positions are still preliminary [23–25]. Reproductive studies of Antarctic octopods have revealed that all species produce large eggs, which likely produce benthic crawling hatchlings that are adult miniatures [26–30]. The embryonic development is estimated to be extremely slow, lasting from months to years and potentially takes up a substantial part of their lifecycles [31–33].

Lifespans of Southern Ocean octopods are unknown, but their life history traits (egg size, fecundity, environmental temperature, adult size) suggest that they live longer than the typical 1–2 years found in temperate and tropical forms [34]. Age estimates in cephalopods can be derived from the quantification of growth increments in hard body parts including statoliths, stylets and gladii (*i*.*e*. vestigial shells), eye lenses and chitinous beaks [35]. Statoliths are calcareous structures typically used to age squids and sepioids, and which growth increments have been validated to be deposited daily [36]. Octopod statoliths lack discernible growth increments and are not suitable for age estimates [37,38]. However, growth increments in beaks and stylets were validated to be formed on an approximately daily basis in octopods living in water temperatures > 17°C [39–42]. The increment periodicity in hard body parts of Antarctic octopods is unknown. Assuming a daily deposition, the quantification of growth increments in *M*. *setebos* stylets suggested a lifespan of about four years [43]. *Pareledone* species have small and delicate stylets which are difficult to prepare for age estimation, and *Adelieledone* lack stylets [17]. Total lifespan in *Pareledone charcoti* (Joubin, 1905) is unknown, but the few specimens kept under laboratory conditions survived up to 21 months and had average daily growth rates of 0.11%, the slowest growth rate ever measured for octopods [44].

While age estimation using stylets is prone to limitations, all cephalopod species have chitinous beaks which also have been used for age determination in squids and octopods [35]. In most cephalopod species the beaks are easy to extract, store and manipulate, and can provide ecological information even when retrieved from predator stomachs [45]. To assess if Antarctic octopod species have longer lifespans than tropical and temperate counterparts the present study investigates size-at-age, growth rates, longevity and maturation of Antarctic incirrate octopods using the quantification of growth increments in beaks.

## Materials and methods

### Collection of specimens

**Fig 1:**
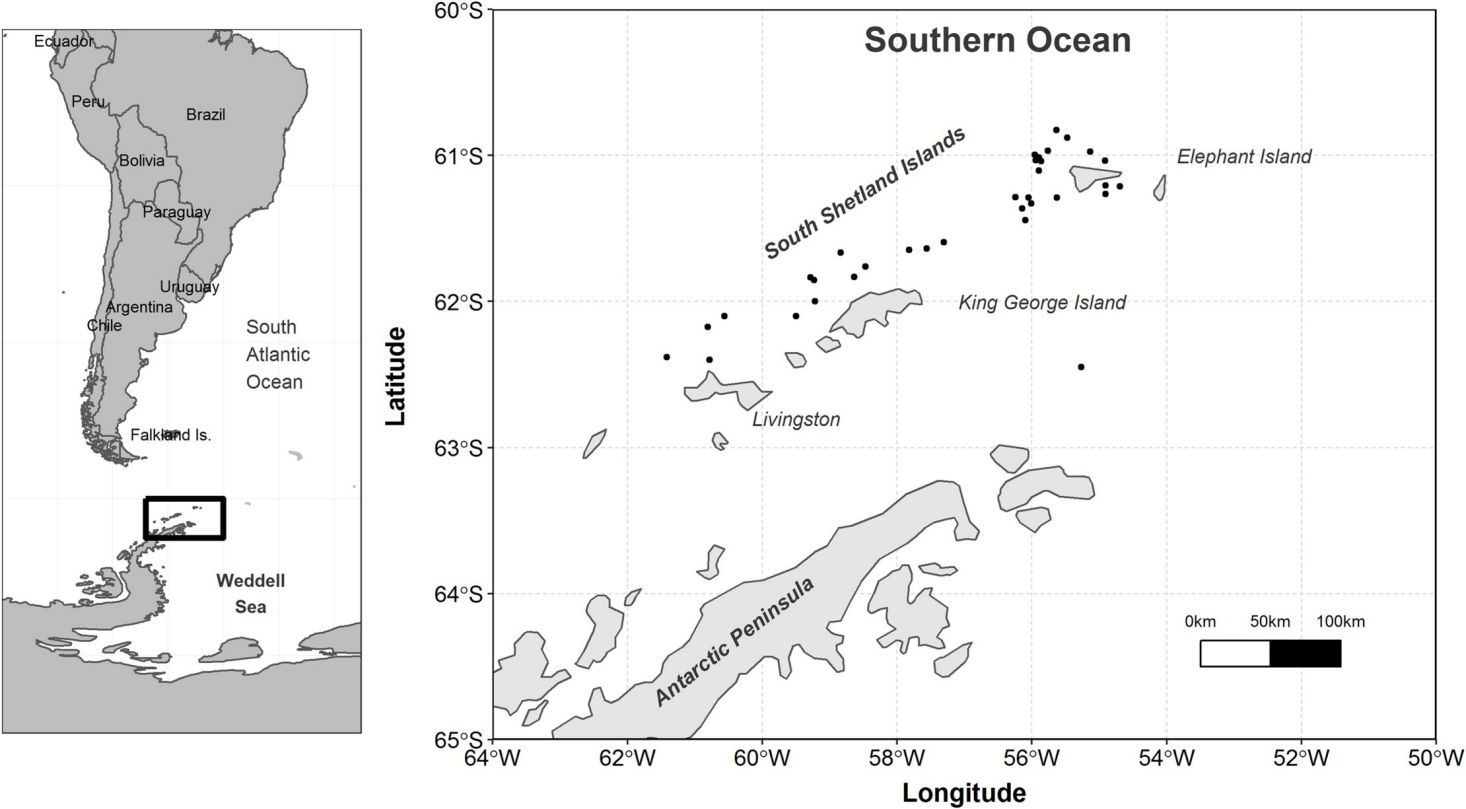
Map of study region. Black dots represent bottom trawl stations (RV POLARSTERN cruise PS79) in the vicinity of the South Shetland Islands in March-April 2012. Sampling depths and biological parameters of octopods collected are summarized in Table **1**.

Species analyzed in this study belong to the Southern Ocean incirrate octopod family Megaleledonidae (two *Adelieledone* spp., eight *Pareledone* spp. and *Megaleledone setebos*) and the family Enteroctopodidae (*Muusoctopus rigbyae*; Vecchione, Allcock, Piatkowski and Strugnell 2009) which all inhabit the continental shelf and slope areas of the Antarctic Peninsula [17,46]. The specimens were during a demersal fish trawl survey in March and April 2012 (RV POLARSTERN cruise PS79-ANTXXVIII/4) [47]. The bottom trawls were conducted at depths between 50-480m north of the Antarctic Peninsula and in the vicinity of the South Shetland Islands (Fig 1). Detailed information on the water temperatures, nets used, fishing depths and geographic position of trawling stations is available in the POLARSTERN 2012 expedition PS79 report [48]. During the cruise, approximately 400 net collected specimens of the species *Pareledone charcoti* were kept alive in aquaria on board and transported to the Alfred Wegener Institute (AWI, Bremerhaven, Germany). The animals were kept alive in the laboratory from the time of their capture during March-April 2012 until April 2013 [49,50]. Eleven of these specimens were used to compare the number of growth increments in the beaks with the time they were kept in captivity, in order to estimate the periodicity of beak increment deposition. The remaining octopod specimens were immediately frozen after sorting and brought to GEOMAR Helmholtz Centre for Ocean Research (Kiel, Germany) where they were stored at -40°C.

In addition, two male specimens of *M*. *setebos* (BM 1598–3858 g) collected during RV POLARSTERN southern Weddell Sea cruises in 2013/2014 [51] and 2015/2016 [52] were also analyzed. These two animals were donated to our project by the AWI. The upper beak from a large male specimen of *M*. *setebos* collected in February of 1989 during a cruise to Halley Bay and Kapp Norvegia [53] was also examined. For this animal only the length was recorded (DML = 210 mm) and its weight was estimated using the allometric equation *BM* = 0.0009*DML*^2.937^, which resulted in a body mass of ~ 6000 g [24]. The three males were included in order to estimate age in large specimens (> 1000 g), since most animals collected in 2012 weighed less than 500 g.

All octopods examined in this study are non-endangered species. All applicable international or institutional guidelines for the care and use of animals were followed. The research was conducted in accordance with the guidelines and ethics of the German law. Sampling during the research expeditions was conducted in accordance with the Commission for the Conservation of Antarctic Marine Living Resources (CCAMLR) [48]. Experiments involving animal husbandry at AWI were approved by the veterinary inspection office (Senatorin für Wissenschaft, Gesundheit und Verbraucherschutz, Bremen, Germany) under the Permit Number AZ: 522-27-11/02-00 (93) [50].

### Summary of specimens

The frozen specimens were thawed in the laboratory and dorsal mantle length (DML, mm), ventral mantle length (VML, mm), total length (TL, mm), body mass (BM, grams), sex and maturity stages were recorded. The species were identified following the descriptions published by Allcock et al. [15–17] and Vecchione et al. [46]. Due to the poor condition, five octopod specimens could not be identified to species level and were not included in the analyses. In total 882 incirrate Antarctic octopods were examined (Table 1).

**Table 1:** Octopod species examined including biological parameters (Sex, DML = dorsal mantle length, BM = body mass, N = specimen numbers, Depth = sampling depth ranges, and Beaks read = number of beaks that were read to quantify growth increments).

Species	Sex	DML (mm) _min-max (avr)_	BM (g) _min-max (avr)_	Depth (m) _min-max (avr)_	N	Beaks read
*Adelieledone piatkowski*	*♂*	62–65 (63)	56–92 (77)	428	3	0
* *	*♀*	68–75 (72)	104–114 (109)	428	2	0
*Adelieledone polymorpha*	*♂*	28–84 (57)	6–132 (63)	155–438 (291)	66	6
* *	*♀*	20–97 (55)	4–201 (63)	100–466 (291)	83	5
	*und*	26–26 (26)	4–4 (4)	207–207 (207)	1	0
*Adelieledone sp*	*♀*	19	1	250	1	0
*Megaleledone setebos*	*♂*	27–230 (82)	11–9760 (1005)	146–880 (321)	32	27
* *	*♀*	27–160 (58)	13–2860 (240)	123–428 (307)	29	23
* *	*und*	210	~6000	782	1	1
*Muusoctopus rigbyae*	*♂*	47–88 (69)	41–320 (136)	62–425 (295)	36	23
* *	*♀*	31–89 (64)	24–337 (133)	62–475 (318)	23	15
*Pareledone aequipapillae*	*♂*	21–78 (44)	4–109 (31)	100–428 (323)	137	82
* *	*♀*	17–64 (43)	2–79 (31)	92–428 (307)	75	51
*Pareledone albimaculata*	*♂*	30–36 (32)	12–18 (15)	275–387 (331)	4	0
	*♀*	33–49 (41)	21–86 (54)	241–241 (241)	2	0
*Pareledone aurata*	*♂*	29–56 (44)	5–47 (32)	127–387 (231)	18	2
* *	*♀*	15–52 (37)	3–41 (23)	100–250 (179)	13	0
*Pareledone charcoti*	*♂*	27–57 (44)	5–49 (28)	62–197 (110)	92	15
* *	*♀*	18–77 (49)	3–124 (46)	62–387 (108)	84	29
*Pareledone cornuta*	*♂*	21–53 (37)	3–56 (29)	170–332 (235)	24	0
* *	*♀*	22–56 (35)	9–60 (27)	127–334 (241)	13	0
*Pareledone felix*	*♂*	22–67 (52)	5–77 (42)	146–438 (310)	38	8
* *	*♀*	21–70 (46)	2–100 (39)	100–438 (251)	23	7
*Pareledone subtilis*	*♂*	38–55 (49)	11–43 (34)	146–197 (162)	6	0
* *	*♀*	31–31 (31)	9–9 (9)	197–197 (197)	1	0
*Pareledone turqueti*	*♂*	6–100 (56)	2–613 (112)	100–475 (248)	40	7
	*♀*	21–116 (60)	6–561 (112)	93–438 (231)	30	4
	*und*	30–30 (30)	12–12 (12)	339–339 (339)	1	0
*Pareledone sp*	*♀*	43–43 (43)	32–32 (32)	279–279 (279)	1	0
* *	*und*	-	19–34 (28)	264–475 (353)	3	0
**Total**				** **	**882**	**305**

♂—male

♀- female

*und*–sex undetermined.

### Reproductive aspects

Comprehensive descriptions of the reproductive system and biology of the species examined in this study (*e*.*g*. morphology and development of gonads, spermatophores and oocytes) have been provided in earlier studies and will not be discussed in detail [16,17,26,29,30,54]. Typically, Southern Ocean octopods have a reproductive system in an advanced stage of development at very small sizes (DML > 20 mm). Males are easily recognizable due to the large sized hectocotylus on the tip of their third right arm. Although females lack distinct external features, juveniles have distinct oviducts and ovaries.

Maturity stages were assessed following an adaptation of the 6 maturity stage scales defined by Vecchione et al. [46] and Daly [54]. For juvenile males in stage II, the whole reproductive system (spermatophoric organ and testis) was weighed. In maturing males (stages III-VI) in which the spermatophoric complex and the testis could be distinguished, the two parts were weighed separately. Spermatophores number and length (SpL) from 160 individuals were also recorded. To examine females for traces of mating activity, the mantle cavity and oviducts were inspected for the presence of spermatophores or spermatangia. The complete female reproductive system (*i*.*e*. ovary, oviducal glands, and oviducts) was removed and weighed. The diameters of the ovaries and the oviducal glands were measured. The ovary was cut open and the oocytes were counted, measured and weighed. The ovaries were also examined for signs of previous spawning events, such as empty follicles and resorbed oocytes. The presence of small oocytes was checked using a dissecting microscope (6.3 to 25x).

For 107 female specimens all the oocytes in the ovary were examined. The oocytes from immature ovaries are spherical (diameter ≤ 2 mm) and unimodal in size. For 47 females with developing and mature ovaries, all oocytes were separated from the connecting epithelial stalks and measured. Oocytes < 4 mm were measured using a stereomicroscope eyepiece graticule; Oocytes > 4 mm were measured using a digital calliper. All weights and measurements were recorded to the nearest 0.1 mg and 0.01 mm. The gonadosomatic index was calculated as: GSI = (GW/BM)*100%, where GW is the total reproductive apparatus weight and BM is the animal’s total weight in grams. Animals were considered mature when GSI > 8% and vitellogenic oocytes were larger than 10 mm [17,54].

### Beak extraction

Thawed specimen beaks were dissected by making an incision on the buccal mass using scissors. The remaining tissues and mucus on the beaks were removed by hand under running tap water and by using cleaning wipes. When necessary, the beaks were submerged for some minutes in diluted sodium hypochlorite (NaCL) and cleaned again. The beaks were soaked in 70% ethanol (to kill bacteria), placed in vials containing distilled water and stored in a refrigerator (4–7°C) until further analysis.

### Age estimation analysis using beaks

**Fig 2:**
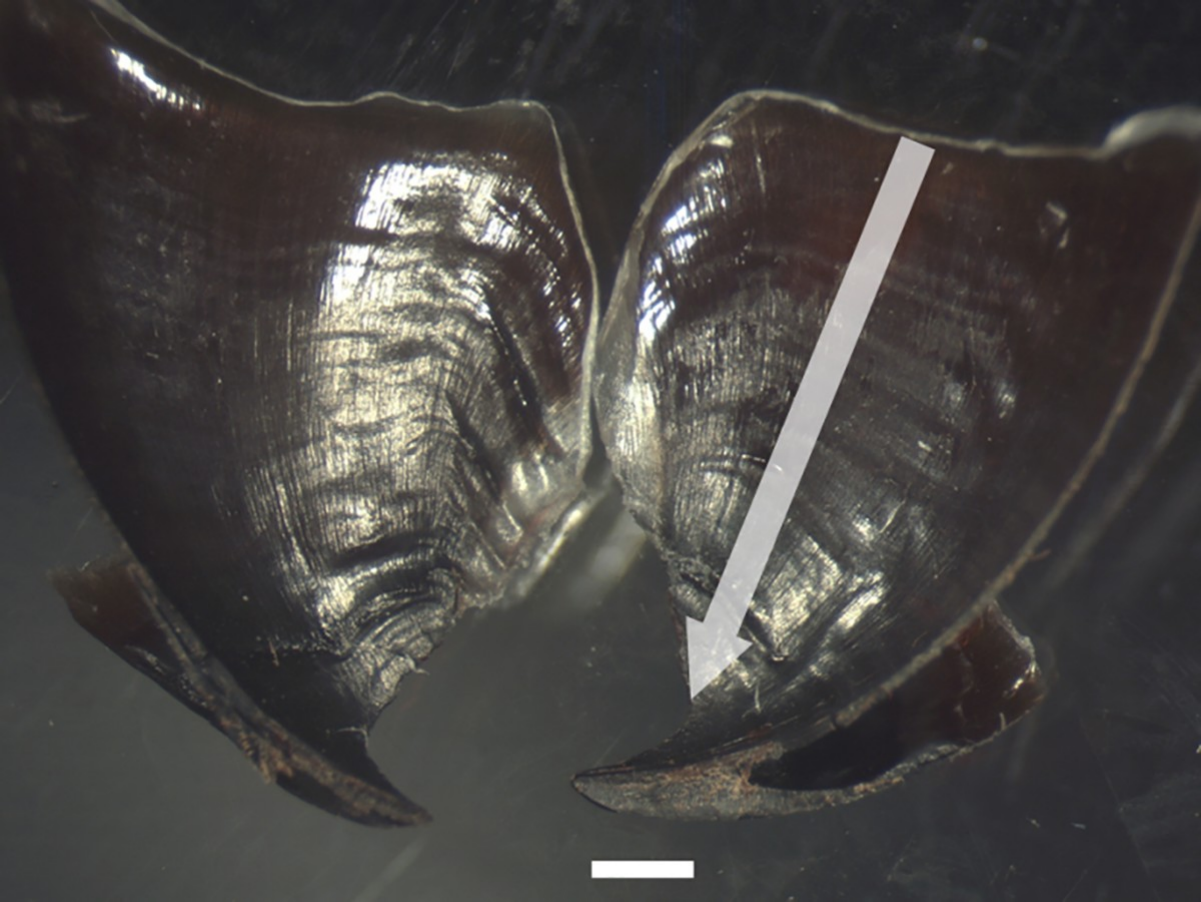
The two halves of a sectioned upper beak from a female of *Pareledone aequipapillae*. Scale bar = 1 mm; The arrow indicates the counting axis.

Procedures for preparing and quantification of beak growth increments followed the methods described by Hernandez-Lopez et al. [55] and Perales-Raya et al. [38]. Beaks were sectioned using scalpels or razor blades in order to obtain two symmetrical halves (Fig 2).

The growth increments were best visualized and counted in the internal part of the upper beaks’ lateral walls surface (LWS). The LWS were examined using a combination of epi-illumination (gooseneck illuminator) and transmitted light. In dark pigmented regions of the beak only reflected light was suitable for increment visualization. The magnification chosen for growth-increment counting ranged between 32 and 50x. Beak hoods were removed for better positioning of the lateral wall surfaces on microscope glass slides and in order to improve light transmission.

**Fig 3:**
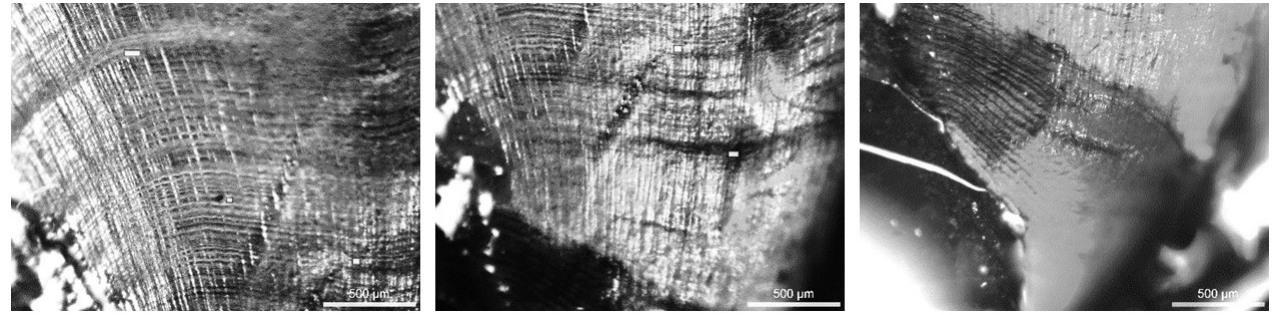
Growth increments in the inner surface of the upper beak lateral wall from a juvenile female stage II of *P*. *charcoti* (DML 43, BM 24 g). Left, posterior region close to the border; Center, medial region of the beak; Right, anterior region close to the rostrum where the first increments were formed, showing check marks in the shoulder region (magnification 50x).

Beaks were placed with their inner surface facing upwards. To prevent dehydration and cracking during increment visualization, the portion of the beak in contact with the microscope slide was kept hydrated with distilled water. Increments were observed via a stereo microscope camera (Leica MZ 9.5) and several (overlapping) photographs were taken in order to cover the entire LWS area (Fig 3). Increment counts and measurements of the distances between growth increments (increment width, μm) were performed with image analysis software (Image Pro Insight). When increment visibility on the anterior region near to the rostrum was poor, an extrapolation was made. The distance of the extrapolated region was divided by the average width of the last counted visible increments.

#### Species selected for age estimation

Four species; *M*. *setebos*, *M*. *rigbyae*, *Pareledone aequipapillae* (Allcock, 2005) and *P*. *charcoti* were selected for age estimation through the observation of growth increments in the upper beak LWS. These species occurred in relatively high numbers (N = 266) and in a variety of sizes and maturity stages.

### Precision of growth increment counting

To obtain the total number of increments in each beak, two counts were performed by a single reader (the first author). The counts were conducted at different occasions. When the increment number differed by more than 10%, a third count was made. If the percentage of agreement did not increase after the third count the beak was excluded from the analysis. Increment count precision was assessed with the coefficient of variation ((CV) [56] and the average percent error (APE) [57]. These terms result from the equations:

$${CV}_{j}=100\%\times \frac{\sqrt{\sum_{i=1}^{R}\frac{{\left(X_{ij}-X_{j}\right)}^{2}}{R-1}}}{X_{j}}$$ (1)

$${APE}_{j}=100\%\times \frac{1}{R}\sum_{i=1}^{R}\frac{\left|X_{ij}-X_{j}\right|}{X_{j}}$$ (2)

where *X*_ij_ is the *i*^th^ age determination of the *j*^th^ beak, *X*_j_ is the mean age estimate of the *j*^th^ beak, and *R* is the number of times each octopus beak was aged. When two age estimates were possible, the age was assumed to be the mean value between counts.

### Growth increment periodicity

For some octopod species, the periodicity of growth increment formation has been validated to occur on a daily basis [40,41]. To test the hypothesis that growth increments are produced on a daily basis in Antarctic octopods, the beaks of specimens of *P*. *charcoti* that were kept in captivity were examined. Eleven female *P*. *charcoti* captured in the 2012 RV POLARSTERN cruise were maintained in aquaria on board and transported to the AWI. The animals were kept in tanks with a re-circulating aquaculture system at 0°C from late March 2012, and were fed one to two times per week with both live and frozen *Crangon crangon* shrimps until they were sacrificed in mid-April 2013 after approximately 12 months [49]. Administration of chemical tracers like oxytetracycline or calcofluor [39,41] in order to evaluate beak increment periodicity was not possible. Unfortunately, we learned about the AWI animals survival time only after Oellermann’s publication in 2015 [50]. The carcasses of the octopods were donated to our study and we compared the approximate husbandry time (~ 12 months) plus the time in the wild, with the number of growth increments in their upper beak LWS.

### Growth

In order to compare growth rates derived from beak growth-increment with those from husbandry experiments [44], we simulated that increments were formed daily. The instantaneous growth rate *G* (% body mass d^-1^) was calculated for each 60-day interval by sex using the equation [58,59]:

$$G=\frac{ln(S_{2})-ln(S_{1})}{\left(t_{2}-t_{1}\right)}.100$$ (3)

where *S*_1_ and *S*_2_ were the average BM (g) at the beginning (t_1_) and end (t_2_) of time interval, respectively.

For the species in which a representative dataset was available, allometric relationships between dorsal mantle length and body mass [*BM =* a*DML*^**b**^], and between body mass and growth-increment number (g*inc*) [*BM* = *a*g*inc*^*b*^] were heuristically fitted using least squares method. All statistical analyses were performed using R version 3.5 and RStudio [60].

## Results

We examined a total of 882 octopod specimens from twelve species belonging to four genera. The most numerous species were *Pareledone aequipapillae* and *Pareledone charcoti* which accounted for 44% of the specimens examined (S1 Fig). The number of species per haul ranged from 1 to 12 ($\bar{x}$ = 4 spp.). The species often overlapped in their spatial and bathymetric distributions in the northern part of the South Shetland Islands, around the Elephant Island and northwest of the Antarctic Peninsula (S2 Fig), and the spatial patterns were similar to those found in previous studies [15–18,46].

### Size distribution and maturity

**Fig 4:**
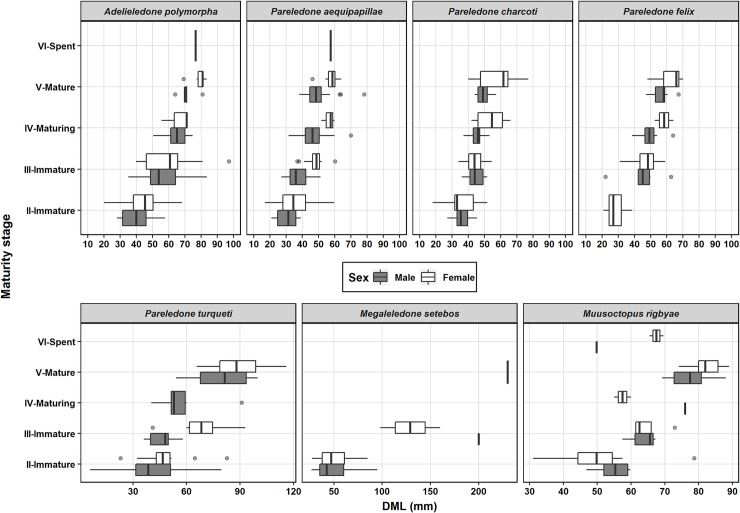
*S*ize distribution at different maturity stages of the most numerous species. Grey circles are outliers.

A wide range of sizes and maturity stages were captured for all species and for both sexes (Fig 4). In ♀ *P*. *charcoti*, ♀ *Megaleledone setebos*, ♀ *M*. *rigbyae* mantle length and body mass frequency distributions did not follow a normal Gaussian distribution, but a multimodal distribution (S3 Fig). Therefore, size differences between sexes were evaluated using the non-parametric Wilcoxon-Mann-Whitney *U*-test. Differences in body mass between sexes were observed only in *P*. *charcoti* (Table 2). For this species the Wilcoxon’s test indicated that females (BM $\bar{x}$ = 45.9 g) weighed on average more than males (BM $\bar{x}$ = 28 g; *U* = 817, *p* < 0.001).

**Table 2:** Results of Mann–Whitney–Wilcoxon test for differences of size or weight between sexes.

Species	Variable	Means $\bar{x}$ (♂ | ♀)	Mann-Whitney test *U*	*p*-value
*A*. *polymorpha*	DML (mm)	56.8 | 55.3	2972	0,37
	BM (g)	62.6 | 63.3	2816	0,77
*M*. *rigbyae*	DML (mm)	68.9 | 64.3	482	0.29
	BM (g)	136 | 133	432	0.78
*M*. *setebos*	DML (mm)	75.3 | 58.1	481	0.49
	BM (g)	890 | 240	471	0.59
*P*. *aequipapillae*	DML (mm)	44.4 | 42.7	5488	0.41
	BM (g)	30.6 | 30.7	5189	0.90
*P*. *charcoti*	DML (mm)	44.0 | 48.7	3013	0.01
	BM (g)	28.0 | 45.9	2132	*< 0*.001*
*P*. *felix*	DML (mm)	51.5 | 45.7	537	0.13
	BM (g)	42.0 | 39.6	481	0.52
*P*. *turqueti*	DML (mm)	56.5 | 59.6	560	0.64
	BM (g)	112 | 112	572	0.74

**Fig 5:**
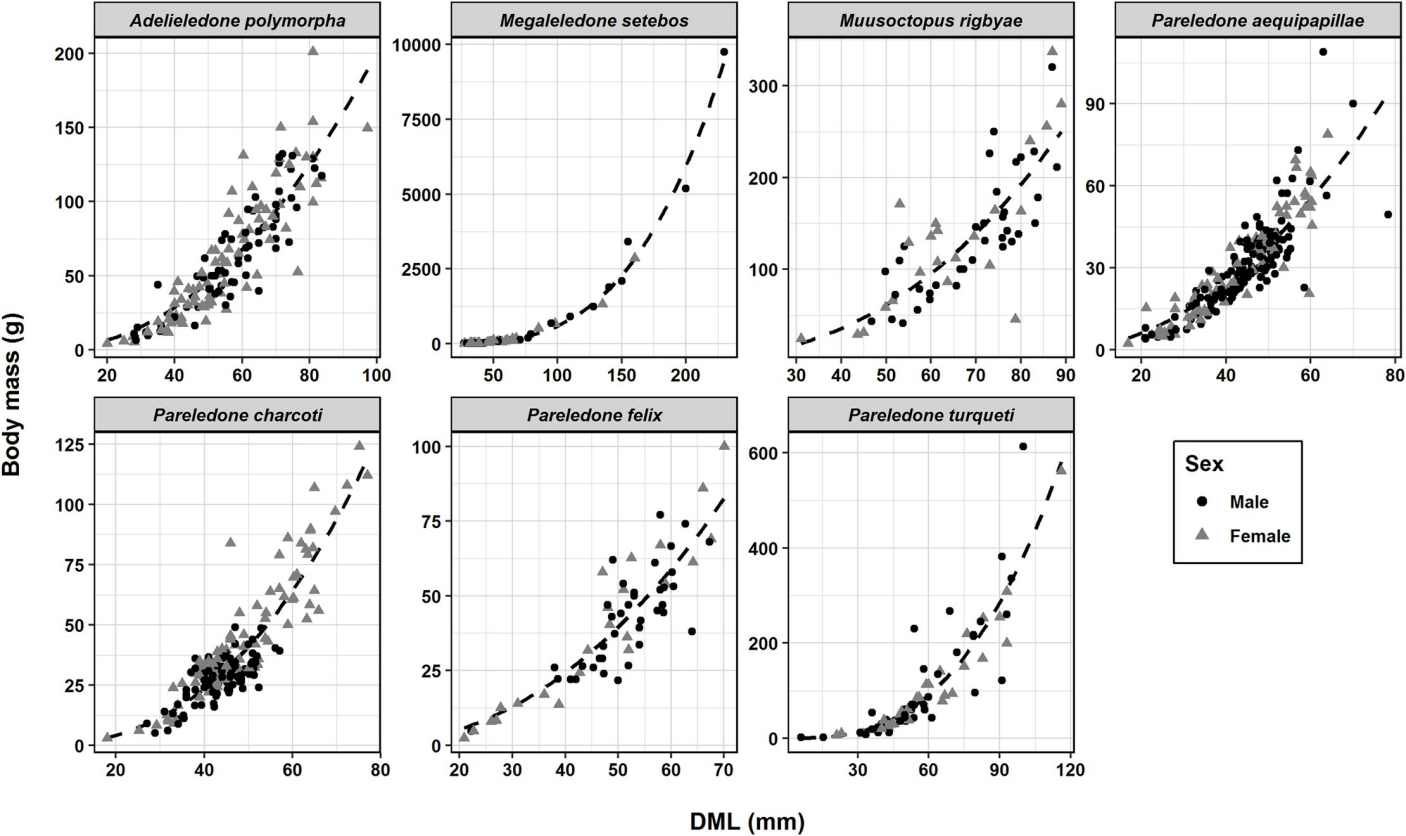
Length-weight relationship of the seven most numerous octopod species. Scale of axes differs between species due to size differences.

Five specimens of *Adelieledone piatkowski* (Allcock, Hochberg, Rodhouse and Thorpe, 2003) were collected during one haul at 428 m, east of King George’s Island. The two females were heavier than the three males (Table 1) and all were immature. For *Adelieledone polymorpha* (Robson, 1930), no sexual size dimorphism was observed (Fig 5).

The largest size range was observed in *M*. *setebos*, with the majority of animals being juveniles (BM 10 to 500 g) and only a few maturing specimens (BM > 1000 g) (Fig 5). *Pareledone turqueti* (Joubin, 1905) and *M*. *rigbyae* were of intermediate size and could reach more than 300 g, but most individuals weighed less than 150 g. Other *Pareledone* species were typically of small size (BM < 200 g) and weighed on average less than 100 g (Fig 5). The relationship between dorsal mantle length and body mass was similar between sexes (S1 Table). In *P*. *charcoti*, males weighed less than females of the same size.

### Description of the beaks

**Fig 6:**
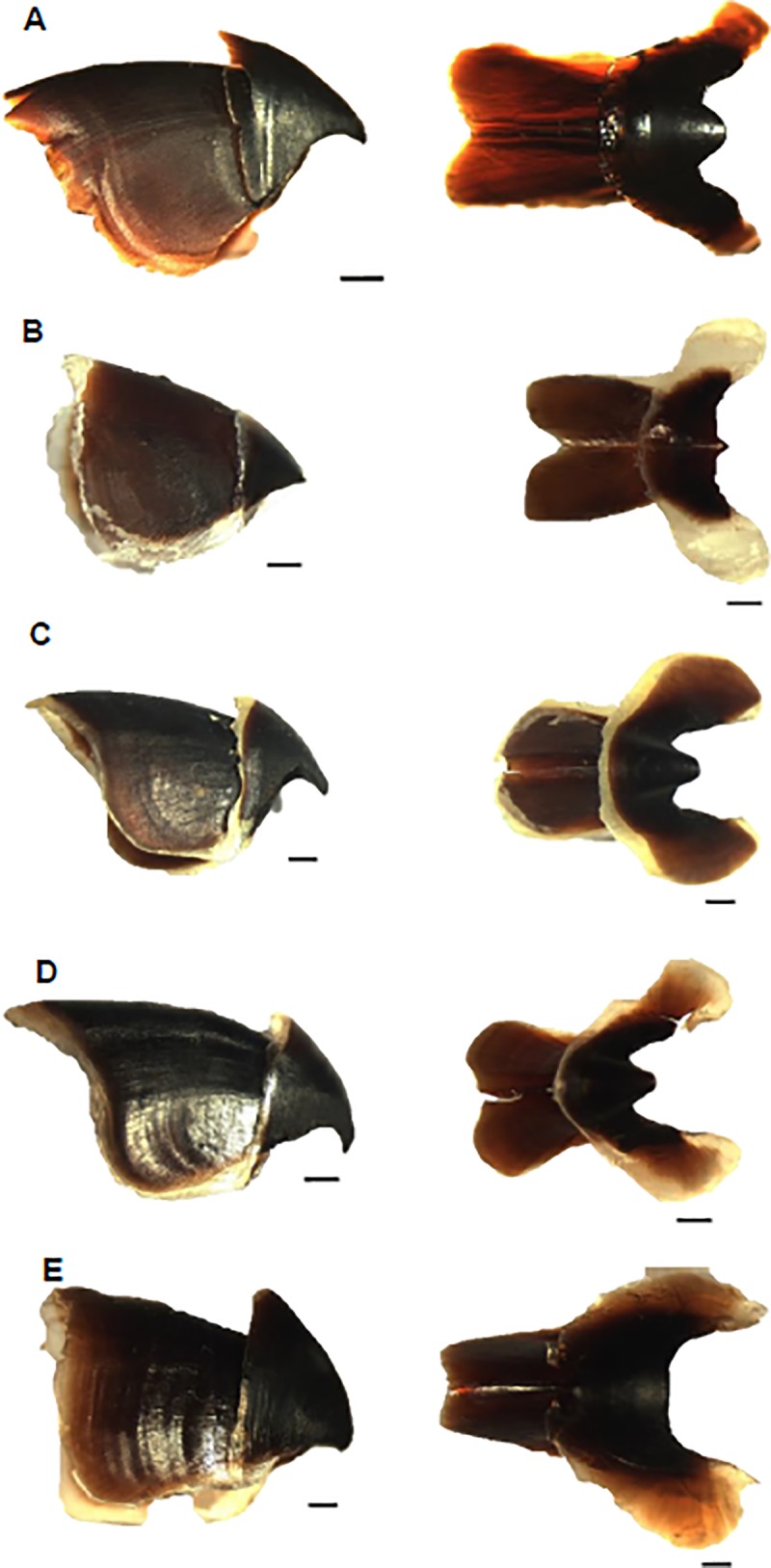
Upper (left) and lower (right) beaks of the five most abundant species of Antarctic octopods analyzed in this study. A—*Muusoctopus rigbyae*; B—*Adelieledone polymorpha*; C—*Pareledone aequipapillae*; D—*Pareledone charcoti*; E–*Megaleledone setebos*. Scale bars represent 1 mm.

The beaks (upper and lower) of the five most abundant species follow the typical octopodid beak morphology (Fig 6, except *Adelieledone* spp. and *M*. *setebos*). All species have robust, darkly pigmented upper and lower beaks with a well-developed hood and curved rostrum with a blunt tip (Fig 6B). The upper beaks of *M*. *setebos* and *A*. *polymorpha* possess a round and shallow projection where the rostrum was supposed to occur, and the crests lack curvature (Fig 6B and 6E). In *Adelieledone* the lower beaks have a characteristic rostrum which is very small, round, sharp and oriented in a straight or upward angle (Fig 6B).

**Fig 7:**
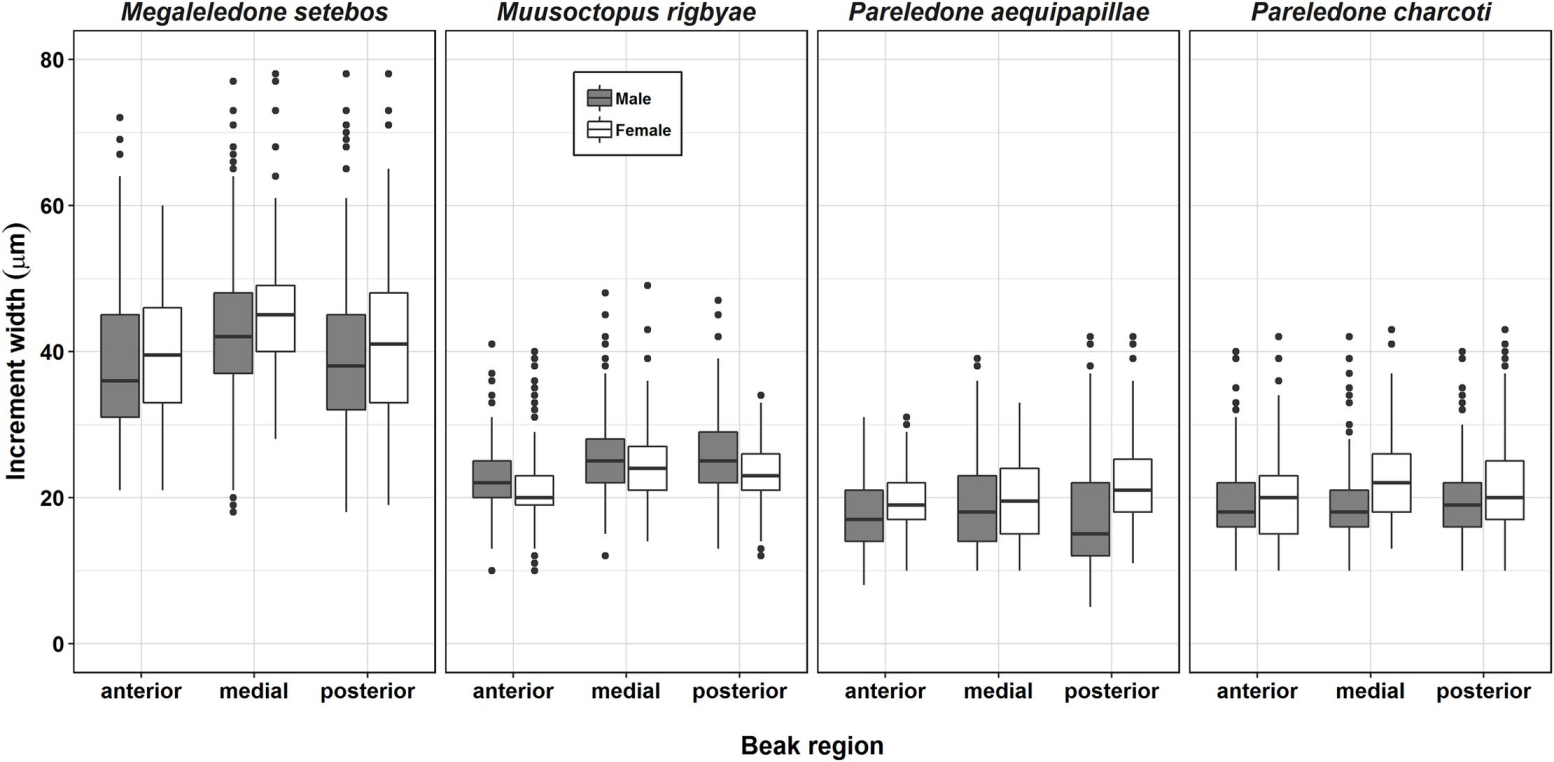
Increment width in different regions of the lateral walls of the octopus beaks analyzed. The anterior region indicates the oldest portion close to the rostrum while the posterior region bear recent formed increments near to the edge of the crest.

The increment width (*W*_*inc*_*)* varied between different species and regions of the beak LWS. In *M*. *setebos* growth increment width ranged from 10 to 78 μm (mean *W*_*inc*_ *=* 40 μm), in *M*. *rigbyae* they are of intermediate size (10–49 μm; $\bar{x}$ = 25 μm), in *P*. *aequipapillae* they ranged from 8 to 42 μm ($\bar{x}$ = 19 μm), while in *P*. *charcoti* they ranged from 6 to 47 μm ($\bar{x}$ = 21 μm). The medial and posterior regions, which are the most recently formed, had higher variability when compared to the anterior region near to the rostrum (Fig 7).

#### Precision of age estimates

For 229 beaks (38 *M*. *rigbyae*, 51 *M*. *setebos*, 96 *P*. *aequipapillae*, 44 *P*. *charcoti*) the counting of growth increments was performed twice. An age bias plot was produced to identify systematic bias between two sets of age estimates from the same beak, using the first count as the reference estimate (S4 Fig). The precision between two counts was higher in *M*. *setebos* compared to other species (CV and APE < 2%). For *P*. *charcoti*, the number of growth increments was higher in the second count. For large individuals of *M*. *rigbyae* the second count was relatively low, resulting in younger age estimates. The resulting coefficients of variation (CV) and average percent errors (APE) between the two counts were less than 5%. The error percentage obtained was considered acceptable (typically < 10% [61]) and it can be assumed that the reader counted the growth increments with good precision. The mean value between two counts was considered for age estimates. For the additional 37 beaks the counts could be performed only once.

### Age estimates and reproductive biology

#### Megaleledone setebos

The size distribution of the 48 *M*. *setebos* from 2012 was biased towards small specimens (DML $\bar{x}$ ♂ 75.3; ♀ 58.1 mm) in the maturity stage II. Most animals weighed less than 500 g, which skewed the results. Beaks of three male specimens (BM 6,000 g, 1,598 g and 3,858 g) that were captured in 1989, 2014, and 2016 were also examined resulting in 51 individuals with increments counted.

Assuming daily deposition of growth increments, age estimates ranged from 98 to 906 days (d). Most males were immature (< stage IV, no spermatophores) and females had small developing oocytes (Table 3). Immature specimens had 98 to 298 increments (BM 10–190 g, $\bar{x}$ = 64 g). In the smallest female (stage II, DML 27 mm, 153 increments), the ovary contained many spherical oocytes (diameter ~ 1mm). Specimens in maturity stage III consisted of one male BM 682 g and two females BM 519 and 670 g, which were estimated 546, 509 and 526 d old, respectively. In four submature males (BM 1882–5190 g) between 671 and 792 increments were counted. Two immature females (stage III, 1326 g and 2860 g) had 678 and 719 increments. The beaks of the largest submature male (BM 9760 g, stage IV) captured during the 2012 cruise were not available for age estimation.

**Table 3:** Summary of reproductive attributes examined from females and males at advanced maturity of different species. DML—dorsal mantle length (mm); BM—body mass (g); GSI—gonadosomatic index; Oocyte N.g^-1^—number of oocytes per gram of BM; Sperm. length—spermatophores length in mm; Sperm. (N)—number of spermatophores per individual; N—specimens examined. Mean values given in parentheses.

**Species** **(females)**	**DML range (mm)**	**BM (g)**	**GSI (%)**	**Number of Oocytes**	**Oocyte Length (mm)**	**Oocyte N.g**^**-1**^	**Oocytes measured N**	**N ind**
*A*. *polymorpha*	55–83 (72)	52–116 (88)	2–13 (7)	38–62 (50)	8–14 (9)	0,2–0,6 (0,4)	100	2
*M*. setebos*	46–68 (61)	43–184 (101)	< 0.5	150–340 (241)	1–2 (1.5)	1,4–5,9 (2,8)	350	7
*M*. *rigbyae*	65–86 (74)	112–256 (167)	1–5 (3)	59–80 (70)	5–15 (12)	0,3–0,5 (0,4)	140	3
*P*. *aequipapillae*	46–60 (57)	29–69 (54)	5–14 (8)	14–40 (25)	2–16 (10)	0,3–1,1 (0,5)	413	17
*P*. *charcoti*	40–77 (58)	28–124 (65)	3–18 (9)	30–94 (55)	1–18 (12)	0,6–2 (1)	302	15
*P*. *felix*	52–70 (64)	61–99 (73)	3–15 (8)	21–22 (21)	5–15 (10)	0,3–0,3 (0,3)	43	2
*P*. *turqueti*	66	78	12	35–35 (35)	3–18 (12)	0,4	35	1
**Species** **(males)**	**DML range (mm)**	**BM (g)**	**GSI (%)**	**Sperm. length range** **(SpL mm)**	**Sperm. (N)**	**N ind**		
*A*. *polymorpha*	50–74 (72)	73–75 (74)	5–5 (5)	28–74 (64)	1–8 (4)	3		
*M*. *rigbyae*	69–84 (76)	110–184 (147)	9–16 (11)	41–144 (69)	5–26 (16)	11		
*P*. *aequipapillae*	36–78 (48)	15–66 (35)	5–11 (8)	22–119 (64)	1–8 (3)	78		
*P*. *charcoti*	43–57 (49)	22–40 (30)	6–10 (8)	23–70 (47)	1–9 (4)	19		
*P*. *felix*	45–67 (54)	26–136 (89)	7–9 (8)	36–72 (53)	1–6 (3)	16		
*P*. *turqueti*	95	336	4	52–92 (72)	2	1		

* Only immature females of *Megaleledone setebos* were observed (stages II and III).

**Fig 8:**
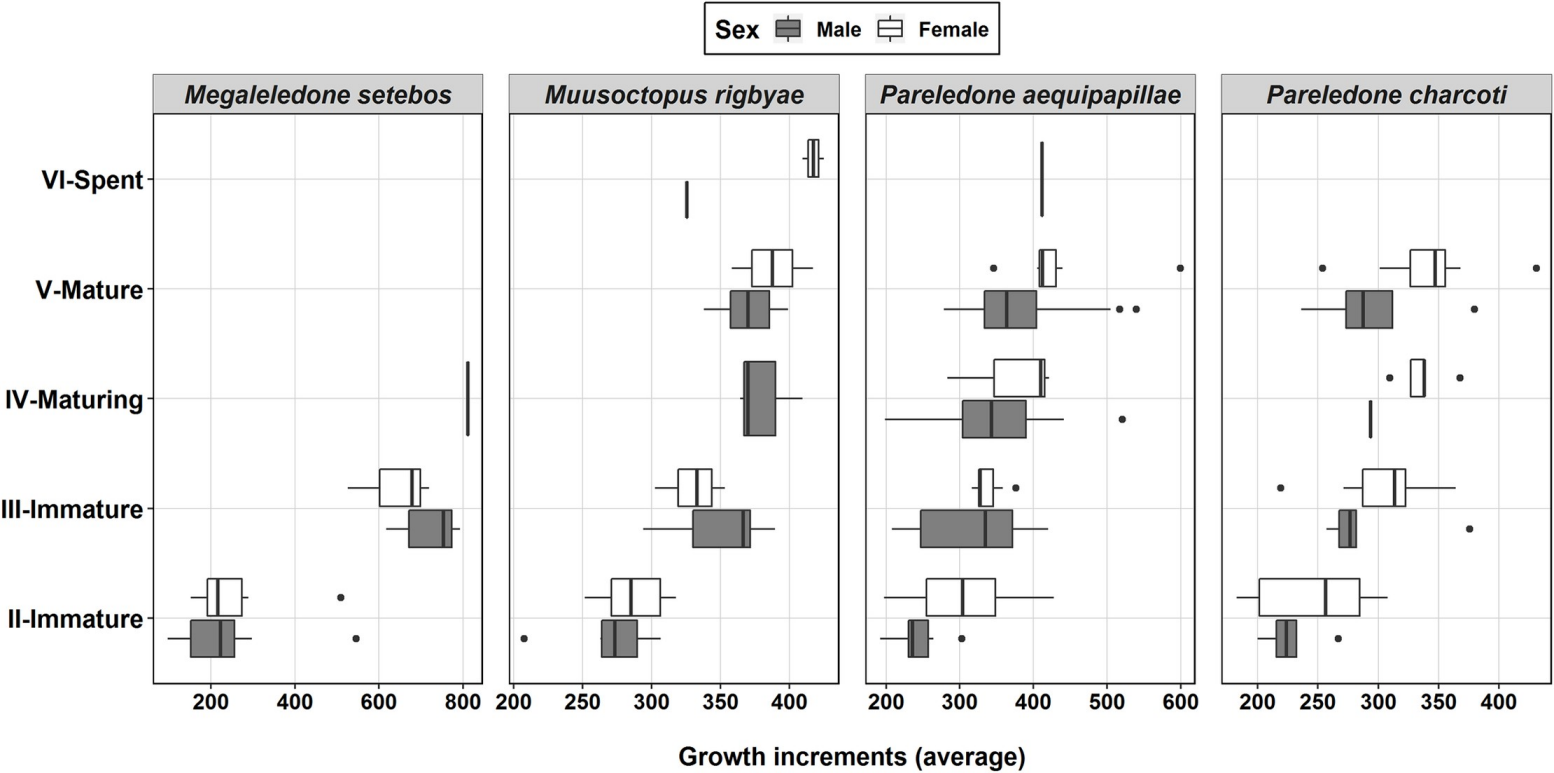
Number of growth increments (mean from two counts) at different maturity stages for the four main species analyzed. Vertical bars indicate presence of only 1 specimen. The largest specimen of *Megaleledone setebos* is not included.

Two male specimens collected in 2014 and 2016 (stage III, DML 172 mm, 1598 g; stage IV, 205 mm, 3858 g) had 628 and 811 increments respectively. The larger specimen had one developed spermatophore (SpL 169 mm) and two more developing. The largest beak examined belonged to a large specimen (sex undetermined; DML 210 mm; ~ 6000 g) captured in the Weddell Sea in 1989 and it had 906 ± 8 increments. Fig 8 shows a box-plot of the range of the number of growth increments at each maturity stage.

#### Muusoctopus rigbyae

*Muusoctopus rigbyae* exhibited a broad range of ages and maturity stages (Fig 8). We examined 59 specimens from 2012 and increments were quantified for 38 (23 ♂, 15 ♀). Increment number in males (DML 46–84 mm, BM 40–184 g) ranged from 207 to 409. Females (DML 31–86 mm, BM 24–256 g) had between 251 and 425 increments. Mature males (> stage IV) had between 325 and 409 increments ($\bar{x}$ = 369), while mature females had between 358 and 425 increments ($\bar{x}$ = 402).

**Fig 9:**
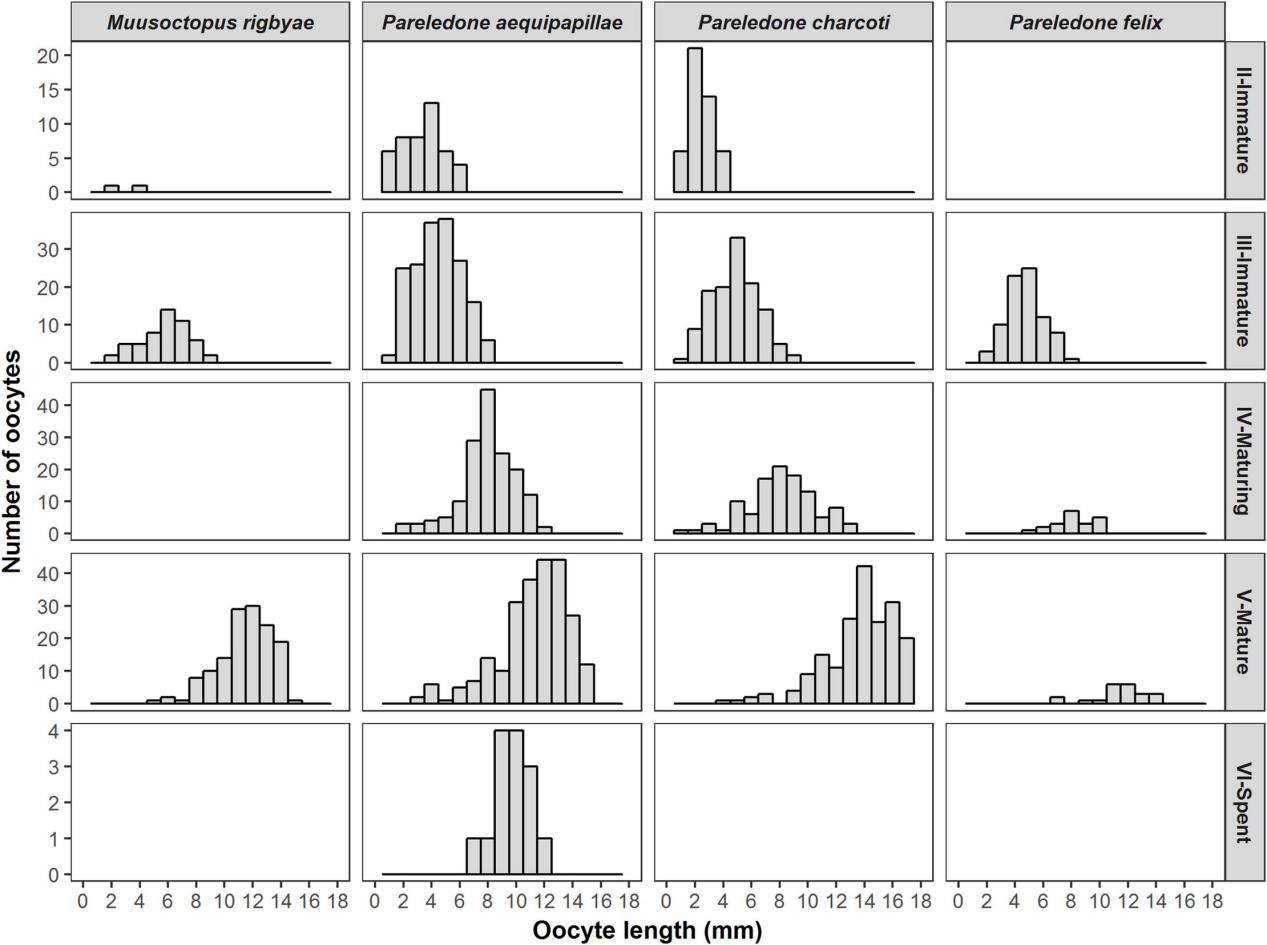
Length frequency distribution of oocytes at different maturity stages for *Muusoctopus rigbyae*, *Pareledone aequipapillae*, *Pareledone charcoti* and *Pareledone felix*.

The ovaries of mature females contained between 59 and 80 oocytes (oocyte length 6 to 15 mm) (Table 3; Fig 9). The two oldest females showed signs of the onset of spawning. One partially spent female (DML 65, BM 112 g, 409 ± 17 increments) had a few fully developed oocytes and some atretic oocytes attached to stalks inside the gonads. The oldest female (DML 69.6, BM 136 g, 425 ± 5 increments) was completely spent with 70 empty follicular folds inside the ovary. There was no evidence of mating (e.g. spermatangia) in either of these females. The males in the most advanced state of maturity (stage V) had 5 to 26 ($\bar{x}$ = 16) fully developed spermatophores (SpL 40–144 mm; $\bar{x}$ = 72 mm). The testis and the spermatophoric complex of one spent male (DML 50, BM 97, 325 ± 24 d) were completely empty. Most of the specimens in advanced maturity had more than 350 growth increments (Fig 8).

#### Pareledone aequipapillae

We examined 212 *Pareledone aequipapillae* (ranging from juveniles to mature) and increments were quantified in 133 of them (82♂, 51 ♀). Males (DML 24–78 mm, BM 4–90 g) had between 192 and 540 increments. Females (DML 16–60 mm, BM 2–69 g) had between 197 and 599 increments. Mature males had between 272 and 540 increments ($\bar{x}$ = 365) while mature females had between 346 and 599 increments ($\bar{x}$ = 433) (Fig 8).

Males had up to eight developed spermatophores (SpL 40–140 mm, $\bar{x}$ = 62 mm) inside the Needham’s sac. Mature females had usually less than 40 oocytes (oocyte length ~ 10 mm, Table 3). A spawning/spent female (DML 57, BM 50 g, 411 ± 38 increments) had 14 follicular yolk oocytes attached to stalks with lengths from 8 to 13 mm ($\bar{x}$ = 10 mm) and 20 empty follicular folds.

#### Pareledone charcoti

The size structure as well as reproductive biology of *P*. *charcoti* was examined in 176 specimens, and the increments in the beaks were counted in 33 of these. Males (DML 28–57 mm, BM 5–40 g) had between 200 and 379 increments, and females (DML 25–77 mm, BM 6–125 g) had between 182 and 431. Mature males (average DML 48 mm and BM 32 g) had between 236 and 379 increments ($\bar{x}$ = 296). Females mature at larger sizes (average DML 60 mm and BM 70 g) and their beaks had between 309 to 431 growth increments ($\bar{x}$ = 355).

One of the largest mature females (DML 77 mm, BM 112 g, 351 ± 1 increments) had a GSI of 18%, and 65 well-developed oocytes (oocyte length 14–18 mm). Female potential fecundity in *P*. *charcoti* is higher than *P*. *aequipapillae* (Table 3). The number of the spermatophores is similar in both species (up to 9, $\bar{x}$ = 4), but they are on average smaller in *P*. *charcoti* (SpL 23–70 mm, $\bar{x}$ = 47).

The beaks of the 11 females that were kept alive at AWI had 219 to 364 growth increments. In spite of the fact that no specific information about the initial size of these animals was provided, all the specimens that were kept in aquaria were measured during the cruise and none of which were hatchlings (N = 250; DML 32–65, $\bar{x}$ = 42 mm; BM 7–119, $\bar{x}$ = 44 g). Six of the AWI specimens were still immature (stage III; DML 35–42 mm, BM 22–33 g), had between 219 and 342 growth increments and their ovaries had between 57 and 75 developing oocytes (< 6.5 mm). The other five females were submature and early mature (DML40-46, BM 28–46) and had between 301 and 364 growth increments in their beak LWS. Their ovaries contained 30 to 62 oocytes (7–15 mm). One female had 49 atretic oocytes (< 5 mm) and 2 large follicular oocytes (length = 10 mm).

### Increment counts in other octopod species

#### Adelieledone polymorpha

The beaks of 13 *A*. *polymorpha* (6 ♂ and 7 ♀) were examined. All females were juveniles (stage II) and increment number ranged from 223 to 304. Submature males (stage IV, DML 50-74mm, BM 62–88 g) had between 218 and 510 growth increments. Their spermatophoric complex contained up to 8 developing spermatophores (SpL 28–74 mm; $\bar{x}$ = 64 mm). Mature females ranged from 55 to 83 mm DML ($\bar{x}$ = 72 mm) with a GSI up to 13%, but age was not estimated. Their ovaries contained 38 to 62 vitellogenic oocytes (diameter = 8–14 mm), but ripe eggs were absent. One spent female (DML 77 mm, BM 52 g) had a flaccid and weakened muscular body and its oviducal glands were pale grey in color, strongly suggesting a senescent state. Inside its ovary, 130 undeveloped oocytes (~ 2.5 mm) were present and connected to epithelial stalks. No vitellogenic oocytes, ripe eggs or post-ovulatory follicles were observed.

#### *Pareledone aurata* (Allcock, 2005)

Two male *P*. *aurata* (stage IV) were examined. The upper beak of the smaller one (DML 40 mm BM 29 g) had between 367 and 470 increments ($\bar{x}$ = 421 ± 54). The larger specimen (DML 43 mm, BM 37 g) had between 371 and 420 increments ($\bar{x}$ = 395 ± 24). However, both individuals had beaks that produced inaccurate counts with a CV higher than 10% and were not suitable for age estimates.

**Pareledone felix** (Allcock, Strugnell, Prodohl, Piatkowski and Vecchione, 2007)

Growth increments were quantified in 15 specimens of *P*. *felix* (8 ♂; 7 ♀). Males (DML 22–54 mm, BM 4–44 g) had between 214 and 331 increments. Females (DML 20–52 mm, BM 2–62 g) had between 198 and 301 increments.

#### Pareledone turqueti

*Pareledone turqueti* is the largest species of the genus, the beaks of seven males (DML 38–100 mm; BM 29–613 g) were examined and exhibited between 245 and 656 increments. Three submature males at stages III and IV (BM 24–35 g) had between 245–328 increments. The four mature males (BM 267–613 g) had 458–656 increments. All four females were immature (stages II-III; DML 32–59, BM 9–113 g) and had 246–459 increments.

### Growth

**Fig 10:**
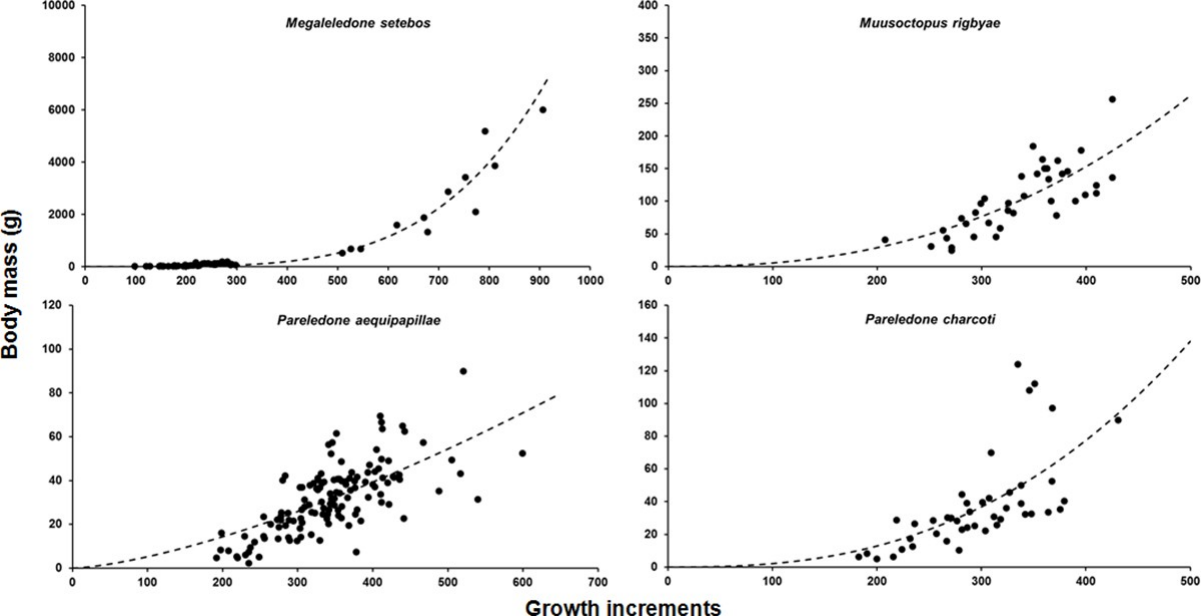
Growth curves showing the relationship between growth increments number (N) and body mass (g) of four species analyzed in this study. Clockwise species order: *M*. *rigbyae*, *P*. *charcoti*, *P*. *aequipapillae* and *M*. *setebos*.

Growth curves were adjusted using the average number of growth increments (g*inc*) in upper beaks LWS and body mass for *M*. *setebos*, *M*. rigbyae, *P*. *aequipapillae* and *P*. *charcoti* (Fig 10). All curves were adjusted for sexes combined (S2 Table). In *M*. *setebos*, the number of growth increments correlates well with the increase in body mass (R^2^ = 0.94). For other species, the correlation is significant but the power curve provided modest fits to the plot (R^2^ = 0.4–0.6).

Assuming a daily periodicity of the growth increments, the giant Antarctic octopus *M*. *setebos* exhibited the highest growth rates (*G*), with values between 0.13 and 2.5% BM d^–1^ (Table 4). *Muusoctopus rigbyae* and *P*. *charcoti* have intermediary *G* values (0.03 to 2.23% BM d^–1^), and *P*. *aequipapillae* had the lowest (0.02 to 1.6% BM d^–1^).

**Table 4:** Instantaneous growth rates (*G*) for four Antarctic octopod species. Avr BM–Average body mass (g) for the age class. Dash indicates data unavailability.

	*Megaleledone setebos*						
	♂				♀		
Age class	N	Avr BM (g)	*G*		N	Avr BM (g)	*G*
90–150	5	14.93	-				-
151–210	5	20.30	0.51		9	29.82	-
211–270	8	93.92	2.55		5	101.52	2.04
271–330	2	128	0.52		5	115.16	0.21
331–390	-	-	-		-	-	-
391–450	-	-	-		-	-	-
451–510	-	-	-		-	-	-
511–570	1	682	0.70		2	594	0.68
571–630	1	1598	1.42		-	-	-
631–690	1	1882	0.27		1	1326	0.67
691–750	-	-	-		1	2860	1.28
751–810	3	3568	0.53		-	-	-
811–870	1	3858	0.13		-	-	-
	*Muusoctopus rigbyae*						
	♂				♀		
Age class	N	Avr BM (g)	*G*		N	Avr BM (g)	*G*
90–150	-	-	-		-	-	-
151–210	1	40.00	-		-	-	-
211–270	2	49.23	0.35		1	31.00	-
271–330	6	74.67	0.69		8	63.63	1.20
331–390	11	134.93	0.99		3	138	1.29
391–450	3	137.33	0.03		3	168	0.33
	*Pareledone aequipapillae*						
	♂				♀		
Age class	N	Avr BM (g)	*G*		N	Avr BM (g)	*G*
90–150	-	-	-	-	-	-	-
151–210	2	6.30	-	-	1	8.40	-
211–270	4	12.80	1.18		8	10.25	0.33
271–330	21	25.11	1.12		15	27.78	1.66
331–390	31	34.80	0.54		17	32.65	0.27
391–450	16	40.14	0.24		9	54.26	0.85
451–510	3	42.27	0.09		-	-	-
511–570	3	54.85	0.43		-	-	-
571–630	-	-	-		1	56.00	0.02
	*Pareledone charcoti*						
	♂				♀		
Age class	N	Avr BM (g)	*G*		N	Avr BM (g)	*G*
90–150	-	-	-		-	-	-
151–210	1	5.10	-		2	7.31	-
211–270	8	19.44	2.23		3	23.28	1.93
271–330	4	30.23	0.74		13	34.60	0.66
331–390	2	37.86	0.38		10	68.10	1.13
391–450	-	-	-		1	89.73	0.46

## Discussion

### Distribution

The occurrence of several *Pareledone* species in the study region corresponds with previous reports that suggested that the South Shetland Islands are a hotspot for speciation of the octopod genus *Pareledone* [62]. Overall, the majority of species were captured between 250 and 350 m bottom depths. The dominance of *Pareledone charcoti* in depths of less than 200 m agrees with Allcock [17], who attributed previous deeper records of this species to potential misidentifications of other *Pareledone* species. [26]. The high abundance of *P*. *charcoti* in shallow waters might be related to a prey preference for amphipods [24]. Amphipods are known to dominate the mobile benthic fauna at ~25 m [19,63]. In the shelf and slope areas of the Weddell Sea, *Pareledone* species including *P*. *charcoti* occur from 200 to 800 m depths, similar to the distribution of amphipod species [14,64]. However, studies on the feeding ecology of Antarctic octopods are scarce [24,54].

*Pareledone turqueti* and *A*. *polymorpha* specimens were collected at a broad range of depths (100–475 m). Both species have circumpolar distribution and occur in depths between 100 m and more than 1000 m [19,65]. Specimens of *Muusoctopus rigbyae* were collected in various depth strata. They were most abundant around 300 m at the Antarctic Peninsula, which is shallower than the typical depths in which this genus occurs in other ocean regions [46]. This characteristic may be related to the evolutionary history of this group, where deep-sea *Muusoctopus* that originated from the northern hemisphere colonized the Southern Ocean five million years ago [46,66]. While specimens of *Megaleledone setebos* are typically found deeper than 250 m, there are shallower records. The type specimen of *M*. *setebos* was collected in a tide pool on Ross Island in 1911 [67]. Further, a large specimen (~ 8 kg) was captured by a SCUBA diver at 20 m in the Davis Sea in 1966 [68]. This broad depth distribution highlights the lack of environmental stratification around the South Shetland Islands shelf and slope, and the eurybathic nature of the studied species. The understanding of the depth distribution of Southern Ocean Octopodidae remains biased as a result of the selected depth strata for trawling.

### Size distribution and maturity

In all examined species, except for *M*. *rigbyae*, maturation advanced gradually with size for both sexes. Vecchione et al. [46] reported several maturity stages over a narrow size range for *M*. *rigbyae* females and suggested that they may mature rapidly after reaching a threshold size. Female biased sexual size dimorphism was observed in *P*. *charcoti*. Among the papillated *Pareledone* species which are typically of small size (BM < 200 g), *P*. *charcoti* females are mature at ~130 g. Specimens of *P*. *turqueti* (BM > 500 g) and *M*. *setebos* (BM > 10,000 g) can attain moderate to large body dimensions, while mature *M*. *rigbyae* and *Adelieledone* spp. are of intermediate size (BM < 500 g).

Size distribution of species described here corresponds with that reported by Barratt [69], who investigated the same group of species from the Antarctic Peninsula (< 1000 m). The animals assigned by Barratt (2009) as *Benthoctopus* cf. *levis* are likely *M*. *rigbyae* [46], since the specimens were captured in the same region of those from our study. The relationship between body mass and dorsal mantle length for seven of the study species correspond with Piatkowski et al. [24]. For *P*. *charcoti* males and both sexes of *M*. *rigbyae*, the length weight curves did not produce a strong fit (r^2^ < 0.7). This may be due to the small sample size and the inclusion of spent individuals (S1 Table). Body weights can decrease dramatically after the release of gametes biasing the correlation between mantle length and weight.

The observed co-occurrence of a broad range of sizes and maturity stages suggests that reproduction and hatching may occur throughout the year. Previous publications already propose a lack of strong seasonal reproductive activity in Southern Ocean incirrate octopods [26,29,70]. Southern Ocean octopods inhabiting waters of -1.8 to 2°C produce relatively large eggs (10–40 mm) which require a longer embryonic development time than species in temperate waters, and likely result in well-developed benthic hatchlings [33,71]. These hatchlings are miniature adults capable of feeding directly on benthic fauna and therefore independent from seasonal plankton blooms [11]. However, most of the Southern Ocean expeditions are restricted to the austral summer (November-April), and sampling efforts throughout the year are necessary in order to elucidate whether or not octopod reproductive activity is coupled with Antarctic seasons.

Similar to Yau et al. [70], who investigated *P*. *turqueti* and *A*. *polymorpha* around South Georgia, we did not find mated, spawning, or brooding females. Their absence could be the result of the species having a nesting behavior in areas that are unsuitable for trawling (*e*.*g*. sponge and rock bottoms). Aggregations of brooding females were observed for *Muusoctopus* and *Graneledone* species in the North Pacific [72] and on a rocky outcrop off Costa Rica in the Central Pacific Ocean [73].

### Beak morphology and growth increments

The diversity of beak morphologies observed in Southern Ocean octopods suggests that the species may occupy different ecological niches. The species examined had the typical Octopodidae beak shapes and may potentially feed on similar prey [17,24,46,54]. The beaks of *M*. *setebos* show the most robust structure. The beaks of *Adelieledone* spp. have an unusual morphology, and lack a distinct rostral tip on the upper beak but they do have a delicate inverted rostrum on the lower beak. Daly and Rodhouse [23] argued that differences in beak morphology between *A*. *polymorpha* and *P*. *turqueti* may indicate differences in foraging and diet. Diet studies showed that both these species prey on crustaceans and polychaetes, but that *P*. *turqueti* in addition feeds on shelled bivalves and gastropods which requires a more robust beak [54].

The increment widths (*W*_*inc*_) in the beak lateral wall surfaces (LWS) of the examined species were on average narrower than previously reported for other octopod species. The maximum *W*_*inc*_ of ~78 μm in *M*. *setebos* is close to the lower limit of the *W*_*inc*_ range observed in the beaks of the temperate *Octopus vulgaris* Cuvier, 1797 (75–100 μm; [38]). The *W*_*inc*_ of 30–50 μm reported for the tropical *Octopus maya* (Voss and Solís, 1966) [40] are similar to those from *M*. *setebos*, but almost two times wider than the average widths observed in other examined Antarctic species (*W*_*inc*_ 19–25 μm). Although growth increments are narrow in Antarctic species, the percentage of agreement between the two age estimates was considered acceptable to this type of analysis, with an average error of less than 7% [61]. The error rates between two increment counts presented here are below those reported by other authors (4–10%) who used similar methods [38,40].

### Age estimates

To date, few studies have investigated age and lifespan in cold water octopods [33,43,74,75]. We applied for the first time the quantification of growth increments in upper beak lateral wall surfaces to estimate age of Antarctic incirrate octopods. Assuming a daily periodicity of increment formation, most of our species reach maturity during the second year of their life. Large specimens of *M*. *setebos* were still immature in their third year of life, and they may have a lifespan of several years. Barratt and Allcock [43] used the quantification of growth increments in the stylet microstructure of the Antarctic *M*. *setebos* and the deep-sea octopus *Bathypolypus sponsalis* to infer lifespans. While the periodicity of stylet increment formation is not validated for these species, their results suggested that the lifespan of *M*. *setebos* is between 3 to 4 years and less than 1 year in *B*. *sponsalis*. The stylet of their largest *M*. *setebos* specimen had 1,077 growth increments (DML 190 mm, BM ~7,000 g) [43]. This estimate is close to the 906 growth increments observed in the beaks of our largest *M*. *setebos* (DML 210 mm, BM ~6,000 g).

For species of intermediate size, the oldest age was inferred to *P*. *turqueti*, with the upper beaks of the most mature male (DML 100, BM 613 g) containing 656 increments. Specimens of *M*. *rigbyae* also seem to reach advanced maturity during their second year, and the oldest female had 425 growth increments. Among the small sized octopods, *P*. *aequipapillae* appears to have the longest lifespan with the most mature animals being older than 500 days. Specimens of *P*. *aequipapillae* and *P*. *charcoti* exhibited a broad range of both size and age for mature animals, which suggests that the final stages of the reproductive activity may take a considerable portion of their lifespan.

Captured immature individuals of *P*. *charcoti* that were kept in aquaria under natural temperature regime (0°C) for more than 12 months had between 219 to 364 beak growth-increments. The beaks of these individuals exhibited the same growth-increment pattern observed in other specimens collected from nature during the expedition, and there were no checks or stress marks in the beak lateral wall surfaces. Although the periodicity of beak-increment formation remains to be properly validated for our study species, the fact that the number of beak increments is lower than the number of days that the animals were kept in captivity strongly suggests that increments in *Pareledone* need more than one day to be formed.

The reduced *W*_*inc*_ of Antarctic species could be related to the relatively slow development of increments under low temperatures. Canali et al. [76] investigated age of *O*. *vulgaris* from the Bay of Naples and observed that recent growth increments in the posterior region of beak lateral wall surfaces were narrower in animals captured during winter months than those captured in summer. The authors argued that the rate of increment formation must be governed by a biological rhythm that depends on metabolic activity, which is mainly influenced by temperature. Perales-Raya et al. [42] developed a model for estimating age of wild *O*. *vulgaris* paralarvae using growth increments from beaks of animals reared at different temperatures. They observed that growth increments were formed daily in animals reared under optimal temperature conditions of 21°C, while in individuals that were kept at ~14°C, one increment was formed every 1.6 days. Their model predicts that at 10°C, a growth increment would be formed every 7^th^ day. Following this model the growth increments in the beaks of Antarctic octopods living at 0°C would very likely require more than one day to be formed, as also suggested by the preliminary validation experiments described here.

Further indirect evidence also supports the hypothesis that growth increments require several days to be formed at low temperatures of the Antarctic Ocean. The instantaneous growth rates (*G*) estimated here of 0.38 to 2.23% BM d^-1^ for *P*. *charcoti* (under the assumption of daily increments) were higher than those observed in animals kept in laboratory (0.08 to 0.13% BM d^-1^) [44]. If the periodicity of beak growth-increment formation is assumed to be 7 days in *P*. *charcoti*, the mature animals examined here would be between four and eight years old. This assumption reduces the estimated *G* to values of 0.05% to 0.32% BM d^-1^, which are closer to the growth rates estimated under lab conditions [44]. A lifespan of about eight years for mature *P*. *charcoti* is congruent with the lifespan estimates derived from life-history traits of octopods living at temperatures below 5°C and observed in situ [33,34]. In this context it is worth noting that there is an anecdotal record of a *P*. *charcoti* specimen that was kept alive for about eight years at the Alfred Wegener Institute (Dr. Felix Mark, *pers*. *comm*.).

In comparison, the quantification of growth increments in stylets of the temperate species *Eledone cirrhosa* (Lamarck, 1798) from the north Atlantic (living at 11–14°C), suggests a maximum lifespan of 516 days with an *G* values of 0.97 to 1.15% BM d^-1^ [77]. Mangold and Boletzky [78] reported a growth rate of 0.9% BM d^-1^ for *O*. *vulgaris* at its lower temperature limit of 10°C. This value is similar to the 0.8% BM d^-1^ found for *Bathypolypus arcticus* (Prosch, 1849) reared at its upper temperature limit of 10°C [75]. Wood [75] also investigated growth in laboratory-reared *B*. *arcticus* at 6°C and estimated that if juveniles continued to grow at the same rate of 0.38% BM d^-1^, they would reach their maturity size of 70 g after 6 years. This was a conservative estimate since animals of this species usually live in waters of 4 ± 2°C, and at this temperature females may brood their eggs for 400 days. Moreover, respiration rates of *P*. *charcoti* are the lowest ever measured for a benthic octopod species. Daly and Peck [44] observed that after 40 days of fasting, respiration rates of one specimen dropped from 10.47 to 9.52 mg O_2_ kg^-1^h^-1^. This respiration value is 1/4 of *E*. *cirrhosa* at 11°C, and 1/8 of *O*. *vulgaris* at 21°C [44].

The above mentioned examples suggest that the growth rates calculated in our study might be substantially overestimated due to the assumption of daily increment formation. Such overestimation is further supported when considering the physiology behind the formation of increments. The time required to complete feeding related processes (e.g. food consumption and digestion) in Antarctic marine invertebrates is two to five times longer than in temperate relatives [79]. Embryogenesis and larval development in Antarctic marine ectotherms are also considerably slower than in temperate species [4,80]. Beak growth results from the metabolic and secretory activity of cells distributed in the buccal mass [81,82]. If all metabolic processes in the organism are delayed under the low temperatures of the Antarctic environment, there is no reason to believe that the process of growth-increment deposition happens at a rate similar to that of species living in temperate waters.

It is also worth noting that because hatchlings are miniatures of the adults, with well-formed beaks, some growth increments may be deposited during the embryonic development. The distinct increments in the anterior rostrum region are potentially formed during the embryonic phase and may result in an overestimation of the adult lifespan. Validation experiments are the next step to determine increment formation in cold water octopod beaks. The administration of oxytetracycline and the injection of calcofluor in husbandry experiments, are successful age validation methods in stylets and beaks respectively [39,41,83] and should also be suitable for experiments on Antarctic octopods which have been successfully kept under laboratory conditions [44,49].

Assuming a daily periodicity for the growth increments in the beaks of Antarctic octopods, females mature during their second year and would require up to 4 years for brooding the eggs at 0°C [33,34]. This assumption would result in a lifespan of about 6 years for the females of intermediate sized species, and more than 7 years for the large *M*. *setebos*. The alternative scenario, where one growth increment represents up to 7 days (i.e. weekly laid), would result in a lifespan of 8–12 years for *M*. *rigbyae* and *Pareledone* spp., and more than 15 years for *M*. *setebos*. The relatively high longevities of Antarctic octopods fit the increased lifespans observed for other marine invertebrates inhabiting Antarctic waters [5,6].

Thorson’s rule [84], a hypothesis that states that there is a positive correlation between egg sizes, embryonic duration, latitude and depth in benthic ectotherms, has been tested and validated for several taxa including octopods [85]. Due to the strong seasonality of plankton production in the Southern Ocean, it may be evolutionary advantageous to produce relatively few but well-developed benthic offspring instead of large numbers of pelagic planktotrophic larvae. Life history theory predicts that the allocation of energy to the production of fewer but larger offspring with extended embryogenesis (or parental care), slow growth rates, and late maturity is found mainly in species with longer lifespans [7,86]. The life history traits of Antarctic octopods resemble those from animals displaying a slower pace of life and longer lifespans.

## Supporting information

S1 Fig Frequency of occurrence of the Antarctic octopod species analyzed. Values inside bars represent the number of animals examined.
(DOCX)

S2 FigDepth distribution of the most numerous species (N > 50) captured during the RV POLARSTERN cruise PS79 in 2012.The dotted line represents the average depth of the bottom trawls ($\bar{x}$ = 255 m). The horizontal black lines inside boxes refer to the median; boxes and whiskers extend from the 25th to the 75th percentile; circles represent outlier cases.(DOCX)

S3 Fig Frequency distribution of dorsal mantle lengths of males and females of the most numerous species analyzed. (DOCX)

S4 Fig Bias plot comparing the precision of two counts of growth increments in the upper beak lateral walls. The dashed line represents the match between the two counts. The coefficient of variation (CV) and average percent error (APE) are presented for each species.
(DOCX)

S1 Table Estimated model parameters of the relationship between mantle length and body mass for seven Antarctic octopod species. The equation is given as BM = *a*DML^*b*^, where BM—body mass (g) and DML—dorsal mantle length (mm). Standard error in parentheses. ♂—male; ♀—female.
(PDF)

S2 Table Estimated model parameters of the relationship between growth-increments number in upper beaks and body mass for four Antarctic octopod species. The equation is given as *BM = a*g*inc*^*b*^, where BM—body mass (g) and g*inc*–growth increments. Standard error in parentheses.
(PDF)
