# Supplementary material for: Life histories of Antarctic incirrate octopods (Cephalopoda: Octopoda)
Source: PLoS One. 2019 Jul 11;14(7):e0219694. doi: 10.1371/journal.pone.0219694 (PMC6622534; doi:10.1371/journal.pone.0219694)
Supplement: S1 Fig — Values inside bars represent the number of animals examined. (DOCX) [file pone.0219694.s001.docx]

**

**

S1 Fig. Frequency of occurrence of the Antarctic octopod species analyzed. Values inside bars represent the number of animals examined.
