# Supplementary material for: Life histories of Antarctic incirrate octopods (Cephalopoda: Octopoda)
Source: PLoS One. 2019 Jul 11;14(7):e0219694. doi: 10.1371/journal.pone.0219694 (PMC6622534; doi:10.1371/journal.pone.0219694)
Supplement: S2 Fig — The dotted line represents the average depth of the bottom trawls (x¯ = 255 m). The horizontal black lines inside boxes refer to the median; boxes and whiskers extend from the 25th to the 75th percentile; circles represent outlier cases. (DOCX) [file pone.0219694.s002.docx]

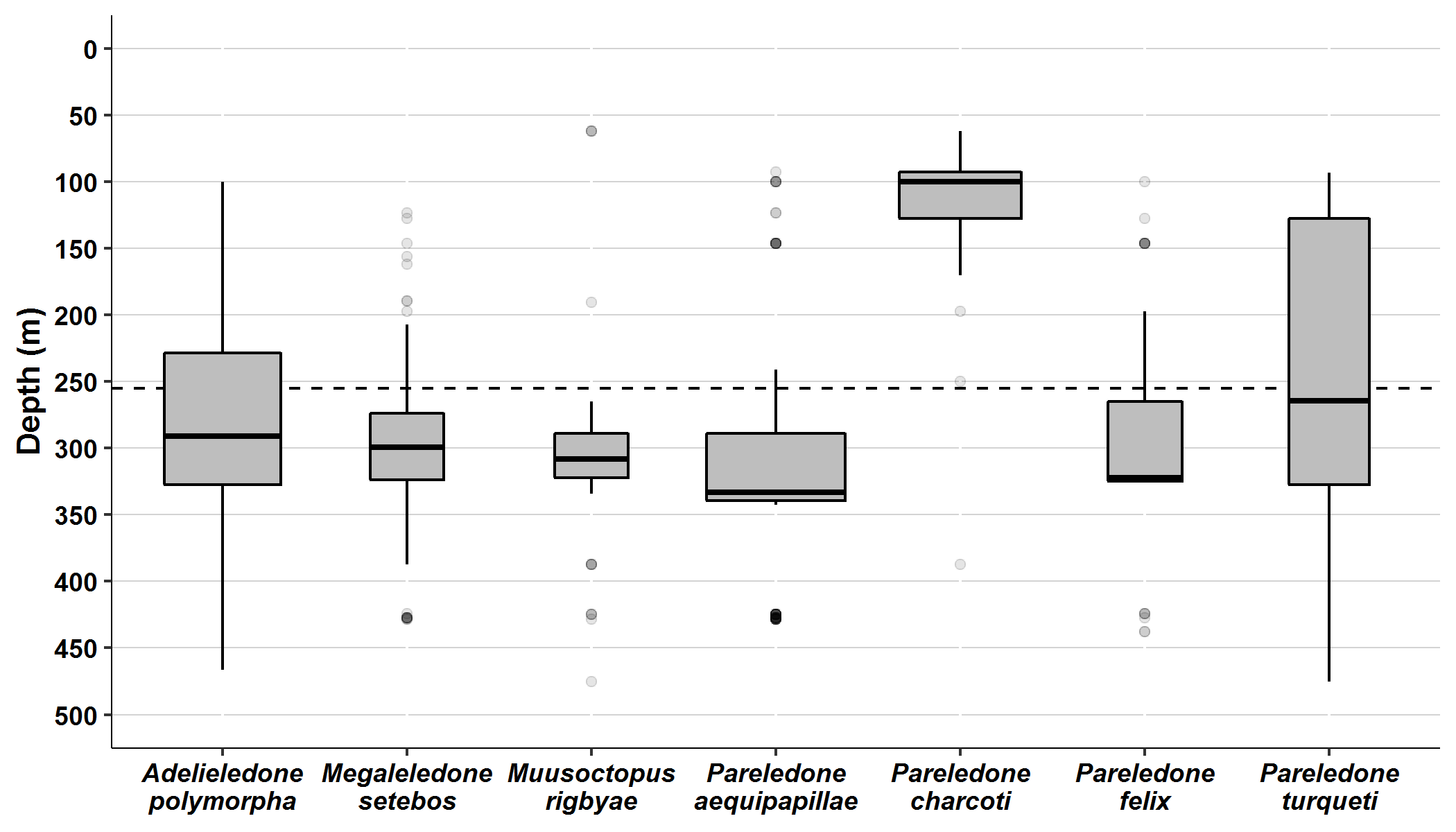


S2 Fig. Depth distribution of the most numerous species (N > 50) captured during the RV POLARSTERN cruise PS79 in 2012. The dotted line represents the average depth of the bottom trawls (*x̅* = 255 m). The horizontal black lines inside boxes refer to the median; boxes and whiskers extend from the 25th to the 75th percentile; circles represent outlier cases.
