# Supplementary material for: Life histories of Antarctic incirrate octopods (Cephalopoda: Octopoda)
Source: PLoS One. 2019 Jul 11;14(7):e0219694. doi: 10.1371/journal.pone.0219694 (PMC6622534; doi:10.1371/journal.pone.0219694)
Supplement: S3 Fig — (DOCX) [file pone.0219694.s003.docx]

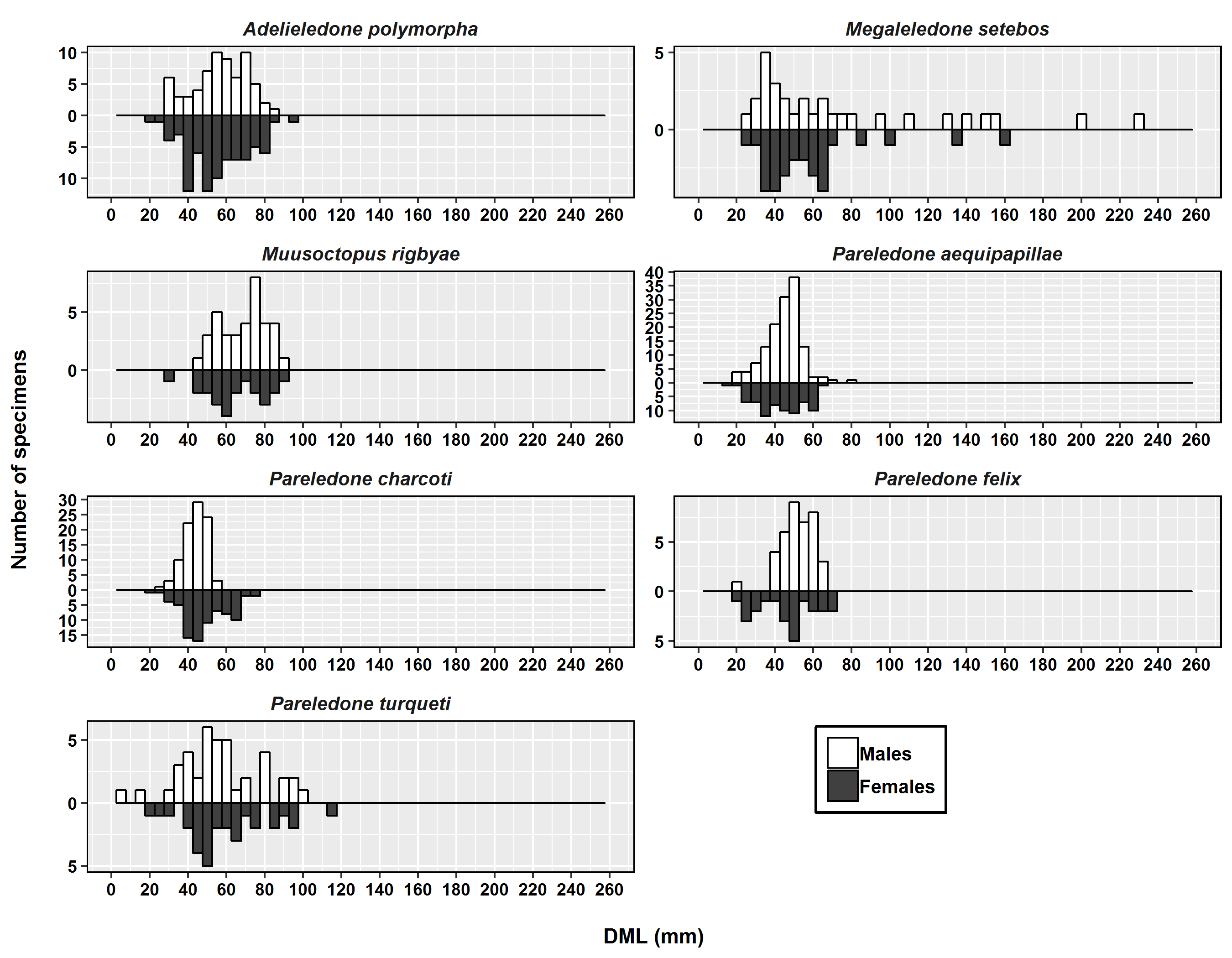


S3 Fig. Frequency distribution of dorsal mantle lengths of males and females of the most numerous species analyzed.
