# Supplementary material for: Life histories of Antarctic incirrate octopods (Cephalopoda: Octopoda)
Source: PLoS One. 2019 Jul 11;14(7):e0219694. doi: 10.1371/journal.pone.0219694 (PMC6622534; doi:10.1371/journal.pone.0219694)
Supplement: S4 Fig — The dashed line represents the match between the two counts. The coefficient of variation (CV) and average percent error (APE) are presented for each species. (DOCX) [file pone.0219694.s004.docx]

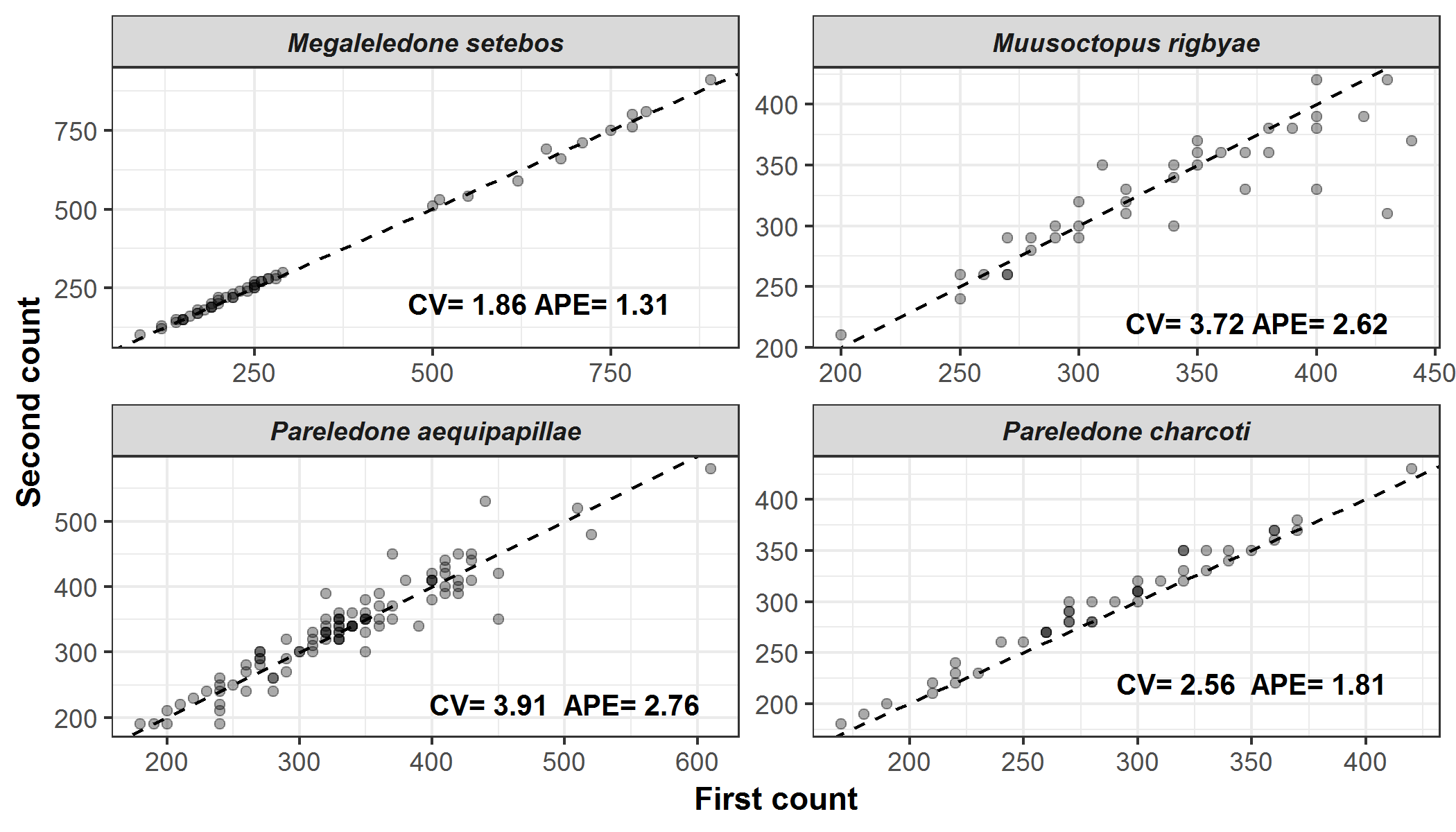


S4 Fig. Bias plot comparing the precision of two counts of growth increments in the upper beak lateral walls. The dashed line represents the match between the two counts. The coefficient of variation (CV) and average percent error (APE) are presented for each species.
