# Supplementary material for: Life histories of Antarctic incirrate octopods (Cephalopoda: Octopoda)
Source: PLoS One. 2019 Jul 11;14(7):e0219694. doi: 10.1371/journal.pone.0219694 (PMC6622534; doi:10.1371/journal.pone.0219694)
Supplement: S1 Table — The equation is given as BM = aDMLb, where BM—body mass (g) and DML—dorsal mantle length (mm). Standard error in parentheses. ♂—male; ♀—female. (PDF) [file pone.0219694.s005.pdf]

S1 Table. Estimated model parameters of the relationship between mantle length and body mass for seven Antarctic octopod species. The equation is given as $BM = aDML^b$, where BM - body mass (g) and DML - dorsal mantle length (mm). Standard error in parentheses. ♂ - male; ♀ - female.

Species	*a*	*b*	*p* value	R^2	N
Adelieledone polymorpha	1.15×10^{-2} (0.01)	2.12 (0.11)	< 0.001	0.77	**149**
♂	6.34×10^{-3} (0.01)	2.24 (0.23)	< 0.001	0.80	66
♀	7.01×10^{-3} (0.00)	2.23 (0.16)	< 0.001	0.75	83
Megaleledone setebos	1.39×10^{-4} (0.00)	3.32 (0.07)	< 0.001	0.98	**59**
♂	9.89×10^{-7} (0.00)	4.23 (0.16)	< 0.001	0.97	30
♀	2.91×10^{-4} (0.00)	3.17 (0.04)	< 0.001	0.98	29
Muusoctopus rigbyae	4.29×10^{-3} (0.01)	2.44 (0.29)	< 0.001	0.63	**59**
♂	9.32×10^{-4} (0.00)	2.79 (0.45)	< 0.001	0.65	36
♀	5.91×10^{-3} (0.01)	2.37 (0.43)	< 0.001	0.62	23
Pareledone aequipapillae	1.48×10^{-2} (0.01)	2.01 (0.09)	< 0.001	0.74	**212**
♂	1.83×10^{-2} (0.01)	1.95 (0.12)	< 0.001	0.67	137
♀	1.04×10^{-2} (0.01)	2.11 (0.16)	< 0.001	0.83	75
Pareledone charcoti	2.61×10^{-3} (0.01)	2.19 (0.20)	< 0.001	0.80	**176**
♂	4.55×10^{-2} (0.04)	1.69 (0.24)	< 0.001	0.54	92
♀	4.75×10^{-3} (0.00)	2.33 (0.09)	< 0.001	0.83	84
Pareledone felix	7.54×10^{-3} (0.01)	2.19 (0.20)	< 0.001	0.76	**61**
♂	1.22×10^{-2} (0.02)	2.05 (0.33)	< 0.001	0.61	38
♀	3.26×10^{-3} (0.00)	2.41 (0.28)	< 0.001	0.88	23
Pareledone turqueti	9.40×10^{-4} (0.00)	2.81 (0.19)	< 0.001	0.82	**70**
♂	4.01×10^{-4} (0.00)	3.01 (0.37)	< 0.001	0.75	40
♀	5.75×10^{-4} (0.00)	2.89 (0.33)	< 0.001	0.94	30
