# Supplementary material for: Life histories of Antarctic incirrate octopods (Cephalopoda: Octopoda)
Source: PLoS One. 2019 Jul 11;14(7):e0219694. doi: 10.1371/journal.pone.0219694 (PMC6622534; doi:10.1371/journal.pone.0219694)
Supplement: S2 Table — The equation is given as BM = agincb, where BM—body mass (g) and ginc–growth increments. Standard error in parentheses. (PDF) [file pone.0219694.s006.pdf]

**S2 Table.** Estimated model parameters of the relationship between growth-increments number in upper beaks and body mass for four Antarctic octopod species. The equation is given as $BM = aginc^b$, where BM - body mass (g) and g*inc* – growth increments. Standard error in parentheses.

| **Species** | ***a*** | ***b*** | ***p* value** | **$R^2$** | **N** |
|---|---|---|---|---|---|
| *Megaleledone setebos* | $9.95 \times 10^{-10}$ (0.00) | 4.34 (0.44) | < 0.001 | 0.94 | 50 |
| *Muusoctopus rigbyae* | $8.38 \times 10^{-5}$ (0.00) | 2.41 (0.41) | < 0.001 | 0.58 | 38 |
| *Pareledone aequipapillae* | $6.45 \times 10^{-3}$ (0.01) | 1.45 (0.15) | < 0.001 | 0.69 | 133 |
| *Pareledone charcoti* | $1.43 \times 10^{-5}$ (0.00) | 2.59 (0.53) | < 0.001 | 0.46 | 44 |
